# Glycolysis drives STING signaling to facilitate dendritic cell antitumor function

**DOI:** 10.1172/JCI166031

**Published:** 2023-04-03

**Authors:** Zhilin Hu, Xiaoyan Yu, Rui Ding, Ben Liu, Chuanjia Gu, Xiu-Wu Pan, Qiaoqiao Han, Yuerong Zhang, Jie Wan, Xin-Gang Cui, Jiayuan Sun, Qiang Zou

**Affiliations:** 1 Department of Immunology, Key Laboratory of Immune Microenvironment and Disease, The School of Basic Medicine, Nanjing Medical University, Nanjing, China.; 2 Shanghai Institute of Immunology, Department of Immunology and Microbiology, National Key Laboratory of Cancer Systems Medicine,; 3 Department of Respiratory Endoscopy, Department of Respiratory and Critical Care Medicine, Shanghai Chest Hospital, and; 4 Department of Urology, Xinhua Hospital, Shanghai Jiao Tong University School of Medicine, Shanghai, China.

**Keywords:** Immunology, Metabolism, Cancer immunotherapy, Glucose metabolism, Innate immunity

## Abstract

Activation of STING signaling in DCs promotes antitumor immunity. Aerobic glycolysis is a metabolic hallmark of activated DCs, but how the glycolytic pathway intersects with STING signaling in tumor-infiltrating DCs remains elusive. Here, we show that glycolysis drives STING signaling to facilitate DC-mediated antitumor immune responses. Tumor-infiltrating DCs exhibited elevated glycolysis, and blockade of glycolysis by DC-specific *Ldha*/*Ldhb* double deletion resulted in defective antitumor immunity. Mechanistically, glycolysis augmented ATP production to boost STING activation and STING-dependent DC antitumor functions. Moreover, DC-intrinsic STING activation accelerated HIF-1α–mediated glycolysis and established a positive feedback loop. Importantly, glycolysis facilitated STING-dependent DC activity in tissue samples from patients with non–small cell lung cancer. Our results provide mechanistic insight into how the crosstalk of glycolytic metabolism and STING signaling enhances DC antitumor activity and can be harnessed to improve cancer therapies.

## Graphical abstract

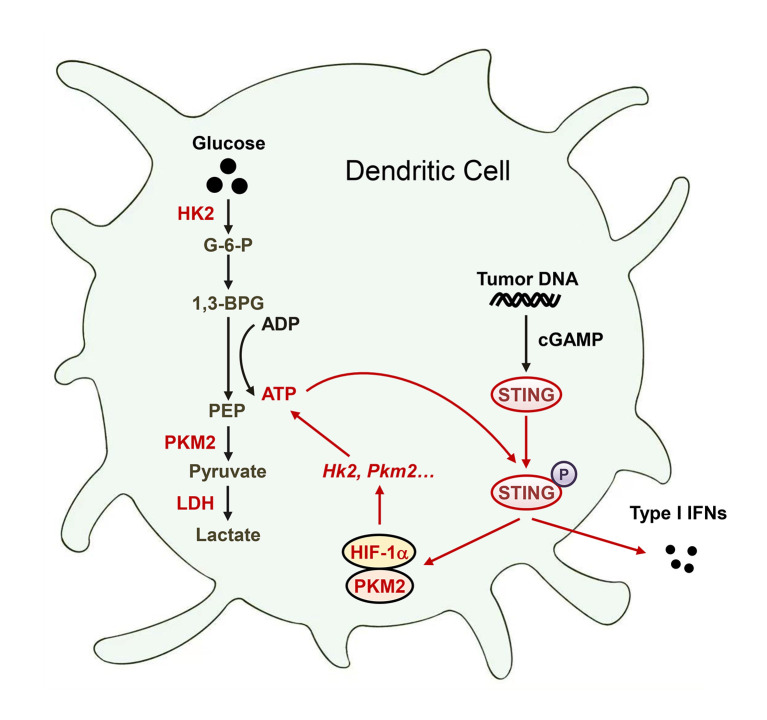

## Introduction

Tumor-infiltrating DCs present tumor antigens and provide cytokine signals to initiate productive antitumor T cell immunity (1, 2). Tumor DNA (Tu-DNA) uptake by intratumoral DCs triggers the activation of the cytoplasmic DNA–sensing cGAS/STING pathway, which is required for type I IFN induction (3–5). This functional STING-dependent cytosolic DNA sensing is critical for spontaneous and radiation-induced antitumor immunity (6–8). Intratumoral injection of STING agonists has been shown to be beneficial for antitumor immunity and tumor immunotherapies in preclinical models (5, 9, 10). However, the intrinsic activation of STING within tumor cells and T cells can promote tumor metastasis and induce T cell death, respectively (11, 12). Thus, therapeutic strategies activating DC-intrinsic STING signaling may hold significant potential for cancer immunotherapy.

Metabolic programs are crucial for DC homeostasis and antitumor function (13, 14). Glucose metabolism serves as an essential metabolic process that maintains cellular energy and cell mass (15). In the absence of oxygen, aerobic glycolysis converts glucose to lactate via lactate dehydrogenase (LDH). LDH has two common subunits, LDHA and LDHB, which combine to generate 5 isoforms (A_4_B_0_, A_3_B_1_, A_2_B_2_, A_1_B_3_, and A_0_B_4_) with distinct kinetic properties (16). In the presence of oxygen, glucose is metabolized into pyruvate, which is imported into mitochondria for the tricarboxylic acid cycle and couples with oxidative phosphorylation (OXPHOS) to generate ATP (15). Immature resting DCs rely on OXPHOS to supply their energy demands (17). In contrast, activated DCs quickly induce glycolysis, and this early glycolytic reprogramming provides important metabolic intermediates to sustain the production of DC activation-related molecules (13, 18). Aerobic glycolysis is a metabolic hallmark of activated DCs. Glucose restriction is predominant in tumors and causes glycolytic and bioenergetic defects in tumor-infiltrating T cells (19). Notably, aerobic glycolysis promotes IFN-γ expression in CD4^+^ T cells through an epigenetic mechanism of *Ifng* transcription (16). Importantly, glycolytic metabolism augments ATP production to drive phosphoinositide 3-kinase signaling, thereby enhancing effector T helper 17 cell and CD8^+^ T cell immunity (20, 21). However, the potential importance of the glycolytic pathway in DC-intrinsic STING signaling and antitumor function in the tumor microenvironment (TME) remains largely unexplored.

Here, we addressed this fundamental question using a combination of metabolomics analysis, conditional gene targeting in mice, and functional cell characterization in mouse tumor models and human cancer tissues. We found that elevated glycolysis augmented glycolytic ATP production in tumor-infiltrating DCs, thus driving STING signaling to facilitate DC-mediated antitumor immune responses. DC-specific deletion of *Ldha*/*Ldhb* blunted STING-dependent type I IFN signaling and dampened antitumor immune responses. Blockade of glycolysis decreased ATP production and caused STING signaling defects, and intracellular ATP delivery restored STING signaling in *Ldha*/*Ldhb*-knockout DCs. DC-intrinsic STING activation accelerated HIF-1α–mediated glycolysis and established a positive feedback loop. Consistent with this finding, the glycolytic pathway was involved with STING-dependent antitumor activity of DCs in tissue samples from patients with non–small cell lung cancer (NSCLC). These results delineate a mechanism by which glycolysis promotes STING-mediated DC antitumor immunity and suggest that enhancing DC-intrinsic glycolysis might help improve the efficacy of DC-centric immunotherapies.

## Results

### STING signaling–activated DCs exhibited enhanced glycolysis.

**Figure 1:**
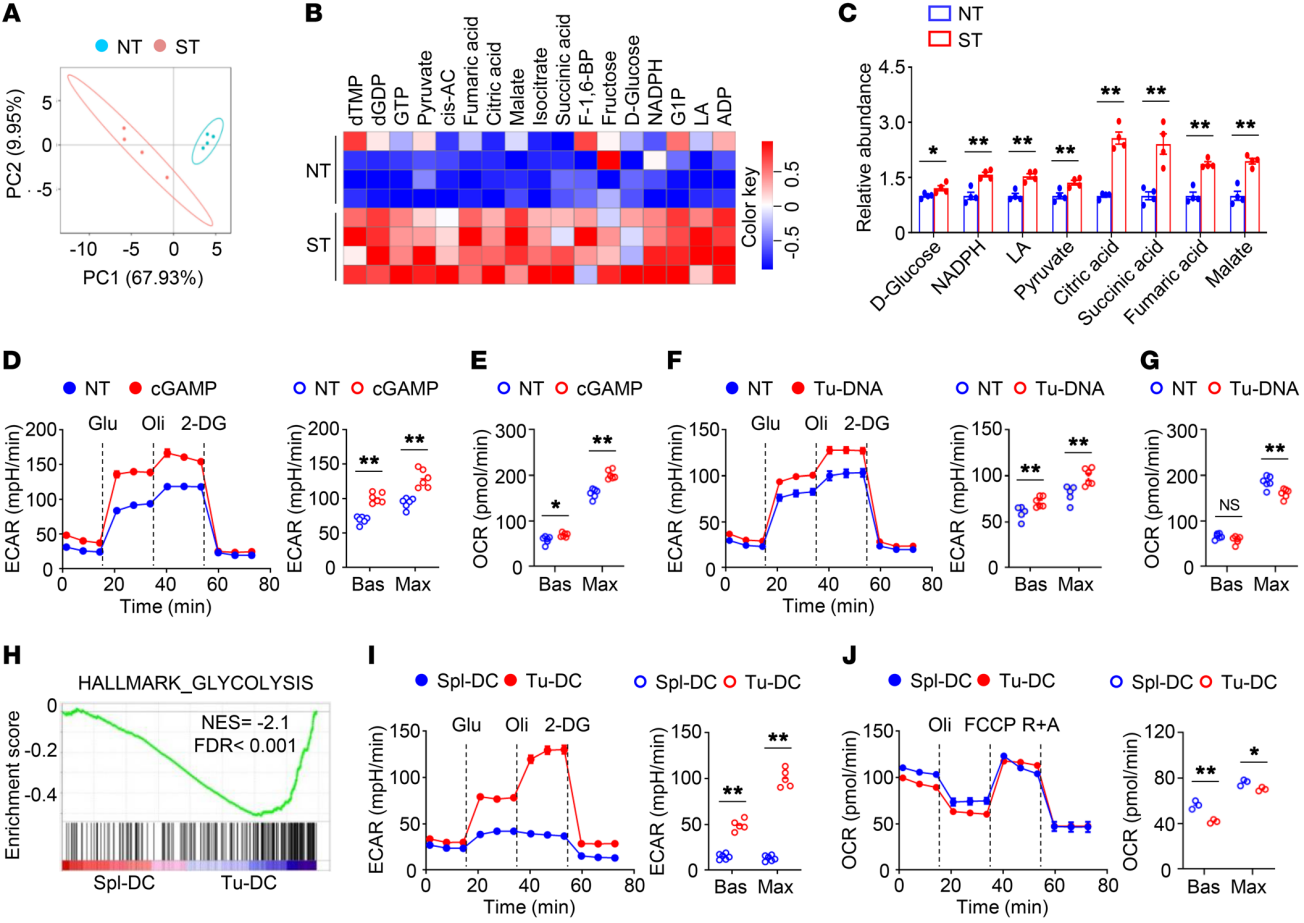
STING signaling–activated DCs exhibit enhanced glycolysis. (**A**) Principal components analysis (PCA) of central carbon metabolome of bone marrow–derived DCs (BMDCs) stimulated with 2 μg/mL 2′3′-cGAMP (cGAMP) for 4 hours (*n =* 4). Each symbol represents data from an individual mouse. NT, nontreated; ST, cGAMP stimulated. (**B** and **C**) Heatmap analysis (**B**) and graph presentation (**C**) of differential metabolites in NT and ST groups from **A**. (**D** and **E**) Extracellular acidification rate (ECAR; *n =* 6; **D**) and oxygen consumption rate (OCR; *n =* 5; **E**) of BMDCs stimulated with 2 μg/mL cGAMP for 4 hours under basal (Bas) or maximum (Max) conditions. (**F** and **G**) ECAR (*n =* 5; **F**) and OCR (*n =* 6; **G**) of BMDCs stimulated with 40 μg/mL tumor DNA (Tu-DNA) for 4 hours under Bas or Max conditions. (**H**) Gene set enrichment analysis of the hallmark glycolysis pathway in the freshly isolated tumor-infiltrating DCs (Tu-DC) compared with that of splenic DCs (Spl-DC). DCs were isolated from MC38 tumor-bearing WT mice on day 14 after tumor injection. (**I** and **J**) ECAR (*n =* 5; **I**) and OCR (*n =* 3; **J**) of splenic and tumor-infiltrating DCs isolated from MC38 tumor-bearing WT mice on day 14 after tumor injection. Representative data are shown from 3 independent experiments in **D**–**G**, **I**, and **J**. Data are shown as the mean ± SEM. Statistical analysis was performed using 2-tailed Student’s *t* test; *P < 0.05; **P < 0.01.

To explore whether glycolytic metabolism involves DC-intrinsic STING signaling, we employed an unbiased, systemic metabolomics approach to examine the glucose metabolic changes during STING agonist cGAMP-induced STING activation in bone marrow–derived DCs (BMDCs) (Figure 1A). Intriguingly, the levels of glycolytic intermediates, such as pyruvate and lactate, were significantly increased in cGAMP-stimulated DCs (Figure 1, B and C). In addition, the levels of tricarboxylic acid intermediates, such as citric acid, succinic acid, fumaric acid, and malate, were also significantly upregulated in cGAMP-stimulated DCs (Figure 1, B and C). Indeed, cGAMP-induced DCs had increased baseline and maximum glycolytic rates (Figure 1D). The OXPHOS rates at baseline and at maximum capacity were also increased in cGAMP-treated DCs (Figure 1E). Upon stimulation with tumor-derived DNA, which activates the cGAS/STING pathway in DCs, the baseline and maximum glycolytic rates were increased and the maximum OXPHOS rates were decreased (Figure 1, F and G). To investigate whether glucose metabolism is reprogrammed in tumor-infiltrating DCs, we performed RNA-Seq using DCs freshly isolated from the spleens and the tumors of tumor-bearing mice. Notably, intratumoral DCs exhibited enrichment (Figure 1H). Consistent with this finding, increased glycolytic activity was observed in tumor-infiltrating DCs (Figure 1I). In contrast, the OXPHOS rates were reduced in tumor-infiltrating DCs (Figure 1J). These results suggest that STING signaling–activated DCs exhibited enhanced glycolysis.

### Blockade of glycolysis inhibits DC antitumor function.

**Figure 2:**
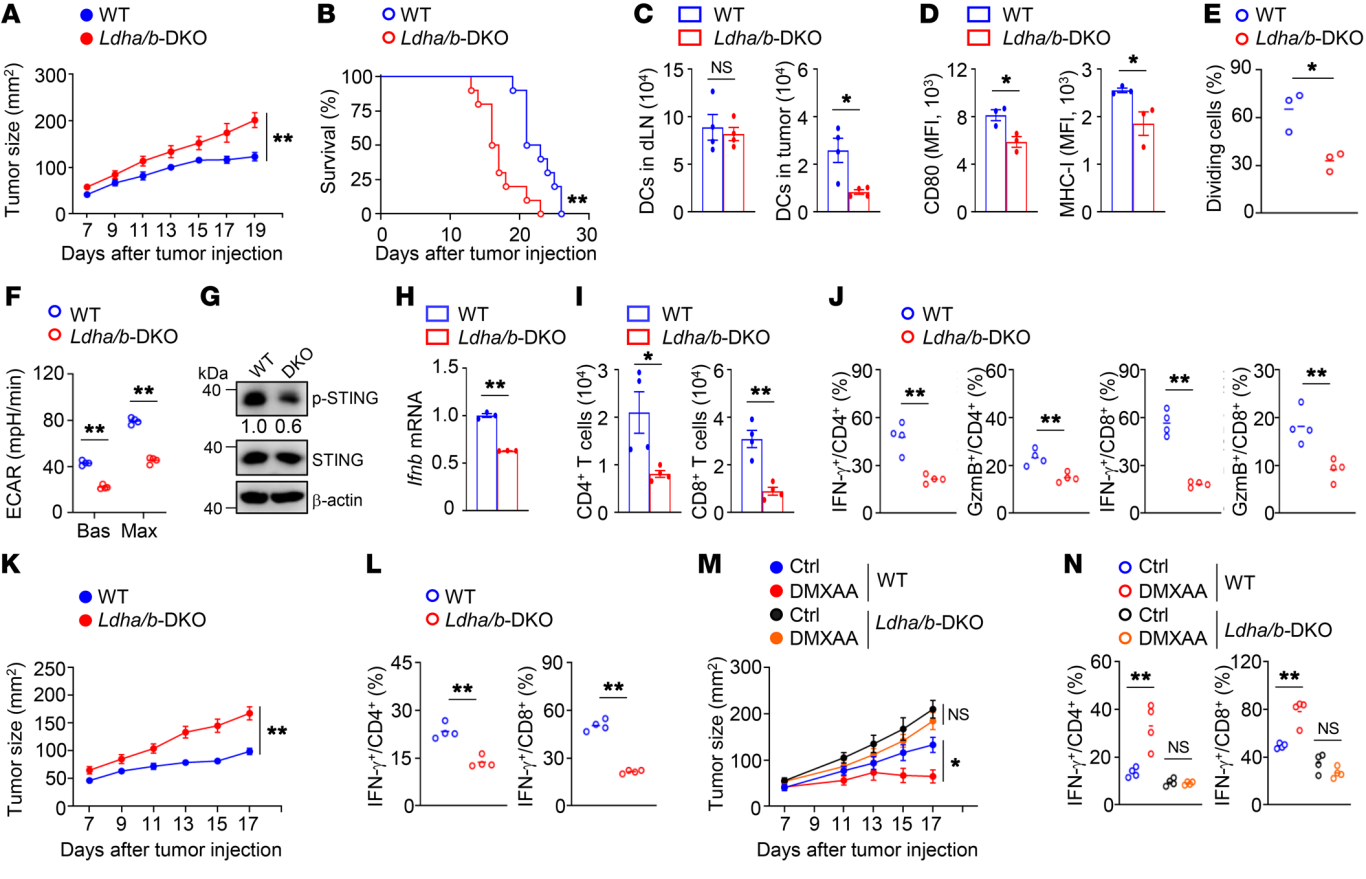
Blockade of glycolysis inhibits DC antitumor function. (**A** and **B**) Tumor growth (*n =* 10; **A**) and survival curves (*n =* 10; **B**) of mice inoculated s.c. with MC38 cells. Representative data shown in **A** and **B** are from different experiments. (**C**) The numbers of DCs in the draining lymph nodes (dLN) and tumors of tumor-bearing mice on day 14 after tumor inoculation (*n =* 4). (**D**) Flow cytometry analysis of CD80 and MHC-I expression in tumor-infiltrating DCs from tumor-bearing mice (*n =* 3). (**E**) Flow cytometric analysis of the division of CTV-labeled OT-I T cells cocultured with tumor-infiltrating DCs (*n =* 3). (**F**) ECAR of tumor-infiltrating DCs from tumor-bearing mice (*n =* 4). (**G**) Immunoblot analysis of tumor-infiltrating DCs from tumor-bearing mice. The numbers indicate the relative densities of indicated protein bands normalized to β-actin. (**H**) qRT-PCR analysis of isolated tumor-infiltrating DCs from tumor-bearing mice. (**I** and **J**) Flow cytometry analysis of T cells from tumor-bearing mice on day 14. (**K**) Tumor growth of MC38 tumor-bearing WT mice transferred with cGAMP-stimulated BMDCs on day 3 after tumor injection (*n =* 9). (**L**) Flow cytometry analysis of tumor-infiltrating T cells of the mice from **K**. (**M**) Tumor growth of mice after i.p. injection with 500 μg DMXAA on day 7 after MC38 tumor injection (*n =* 7). Ctrl, without DMXAA injection. (**N**) Flow cytometry analysis of tumor-infiltrating T cells of the mice from **M**. Representative data are shown from 3 independent experiments. Data are shown as the mean ± SEM. Statistical analysis was performed using 2-way ANOVA (**A**, **K**, and **M**), log-rank (Mantel-Cox) test (**B**), 1-way ANOVA (**N**), and 2-tailed Student’s *t* test (**C**–**F**, **H**–**J**, and **L**); *P < 0.05; **P < 0.01.

To explore the role of aerobic glycolysis in DC antitumor activity, *Ldha*-floxed mice and *Ldhb*-floxed mice were crossed with *Cd11c*-Cre mice to obtain *Ldha*^fl/fl^*Ldhb*^fl/fl^ (designated as WT) and *Ldha*^fl/fl^*Ldhb*^fl/fl^ *Cd11c*-Cre (designated as double-knockout [*Ldha/b*-DKO]) mice (Supplemental Figure 1, A–C; supplemental material available online with this article; https://doi.org/10.1172/JCI166031DS1). Six-week-old WT mice and *Ldha*/*b*-DKO mice had similar frequencies of DCs in the bone marrow and spleen (Supplemental Figure 1, D–H). In addition, LDHA/LDHB deficiency did not alter the frequencies of T cells in the thymus and spleen or peripheral T cell homeostasis in 6-week-old mice (Supplemental Figure 2, A–D). Notably, *Ldha*/*b*-DKO mice displayed a profound increase in MC38 tumor growth with premature lethality (Figure 2, A and B). The numbers of DCs in the draining lymph nodes were similar between tumor-bearing WT mice and tumor-bearing *Ldha*/*b*-DKO mice (Figure 2C). Importantly, LDHA/LDHB deficiency reduced the numbers of, the expression of CD80 and MHC-I in, and the antigen cross-presentation ability of tumor-infiltrating DCs (Figure 2, C–E). Moreover, the tumor-bearing *Ldha*/*b*-DKO DCs displayed decreased glycolytic rates, reduced phosphorylation of STING, and impaired type I IFN induction (Figure 2, F–H). *Ldha*/*b*-DKO mice exhibited decreased numbers of tumor-infiltrating CD4^+^ and CD8^+^ T cells and decreased frequencies of tumor-infiltrating IFN-γ–producing and granzyme B–producing CD4^+^ and CD8^+^ effector T cells (Figure 2, I and J). Furthermore, LDHA/LDHB deficiency also diminished glycolysis and STING activation in tumor-infiltrating DCs and impaired DC antitumor immunity in B16-F10 melanoma model. (Supplemental Figure 3, A–H). We used an animal model of BMDC-based therapy to confirm the importance of LDHA/LDHB in DC antitumor functions. After inoculation of WT mice with MC38 tumor cells, we injected cGAMP-treated WT BMDCs or LDHA/LDHB-deficient BMDCs into the tumor-bearing mice. Compared with the WT BMDCs, the LDHA/LDHB-deficient BMDCs were less potent in suppressing tumor growth and inducing tumor-infiltrating IFN-γ–producing T cells (Figure 2, K and L). We further treated tumor-bearing WT and *Ldha*/*b*-DKO mice with the STING agonist DMXAA. DMXAA treatment effectively suppressed tumor growth and induced tumor-infiltrating IFN-γ–producing T cells in the tumor-bearing WT mice (Figure 2, M and N). However, DMXAA treatment had a negligible effect on the tumor growth and the induction of IFN-γ–producing T cells in the tumor-bearing *Ldha*/*b*-DKO mice (Figure 2, M and N). These data suggest that blockade of LDHA/LDHB-mediated aerobic glycolysis suppresses DC-mediated antitumor immune responses.

### Glycolysis drives STING signaling in DCs.

**Figure 3:**
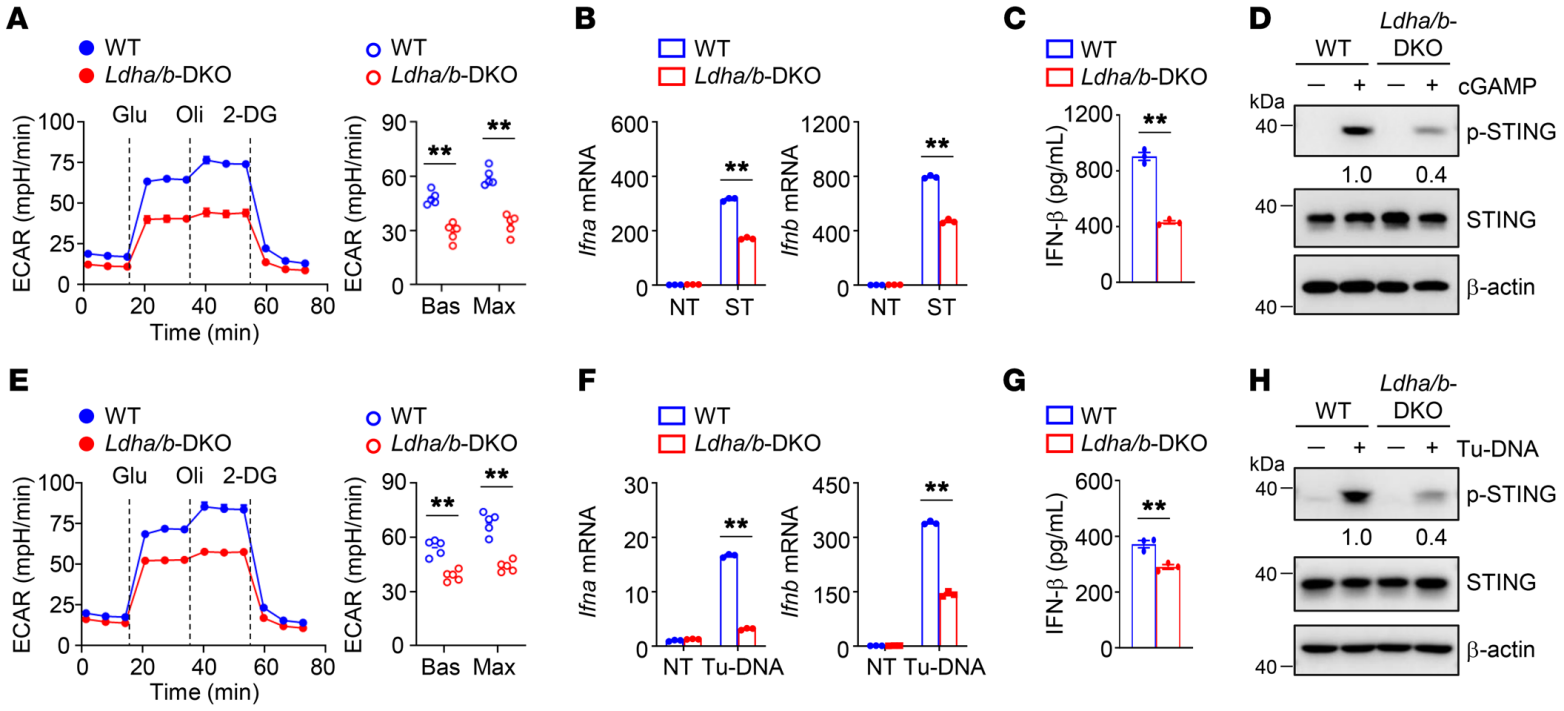
Glycolysis drives STING signaling in DCs. (**A**) ECAR of WT and *Ldha/b*-DKO BMDCs stimulated with 2 μg/mL cGAMP for 4 hours under Bas or Max conditions (*n =* 5). (**B**) Relative mRNA expression level of WT and *Ldha/b*-DKO BMDCs stimulated with 2 μg/mL cGAMP for 3 hours was determined by qRT-PCR. (**C**) IFN-β protein level of WT and *Ldha/b*-DKO BMDCs stimulated with 2 μg/mL cGAMP for 8 hours was determined by ELISA. (**D**) Immunoblot analysis of indicated proteins in whole-cell lysates of BMDCs stimulated with 2 μg/mL cGAMP for 4 hours. (**E**) ECAR of WT and *Ldha/b*-DKO BMDCs stimulated with 40 μg/mL Tu-DNA for 4 hours under Bas or Max conditions (*n =* 5). (**F**) Relative mRNA expression level of WT and *Ldha/b*-DKO BMDCs stimulated with 40 μg/mL Tu-DNA for 3 hours was determined by qRT-PCR. (**G**) IFN-β protein level of WT and *Ldha/b*-DKO BMDCs stimulated with 40 μg/mL Tu-DNA for 8 hours was determined by ELISA. (**H**) Immunoblot analysis of indicated proteins in whole-cell lysates of BMDCs stimulated with 40 μg/mL Tu-DNA for 4 hours. The numbers indicate the relative densities of indicated protein bands normalized to β-actin. Representative data are shown from 3 independent experiments. Data are shown as the mean ± SEM. Statistical analysis was performed using 2-tailed Student’s *t* test; **P < 0.01.

To define the importance of glycolysis in STING signaling events, we activated DCs in the presence or absence of the glycolysis inhibitor 2-deoxy-D-glucose (2-DG). After the addition of 2-DG, the induction of type I IFNs was greatly diminished in cGAMP-stimulated DCs (Supplemental Figure 4, A and B). Parallel experiments revealed that inhibition of glycolysis by another glycolysis inhibitor, either sodium oxamate (OXA) or dichloroacetic acid (DCA), also attenuated the induction of type I IFNs in activated DCs (Supplemental Figure 4, C–G). Moreover, cGAMP-induced STING activation, as shown by its phosphorylation at Ser365, was abolished in glycolysis inhibitor-treated DCs (Supplemental Figure 4, H–J). Importantly, upon cGAMP activation, the glycolytic rates were largely decreased in *Ldha*/*b*-DKO DCs (Figure 3A). In addition, the induction of type I IFNs and the phosphorylation of STING were reduced in cGAMP-stimulated *Ldha*/*b*-DKO DCs (Figure 3, B–D). Consistent with these results, upon stimulation with Tu-DNA, LDHA/LDHB deficiency reduced the glycolytic rates, type I IFN induction, and STING phosphorylation (Figure 3, E–H). These data suggest that inhibition of glycolysis in DCs blunts STING-dependent type I IFN responses.

### Glycolysis potentiates STING-dependent DC antitumor functions.

**Figure 4:**
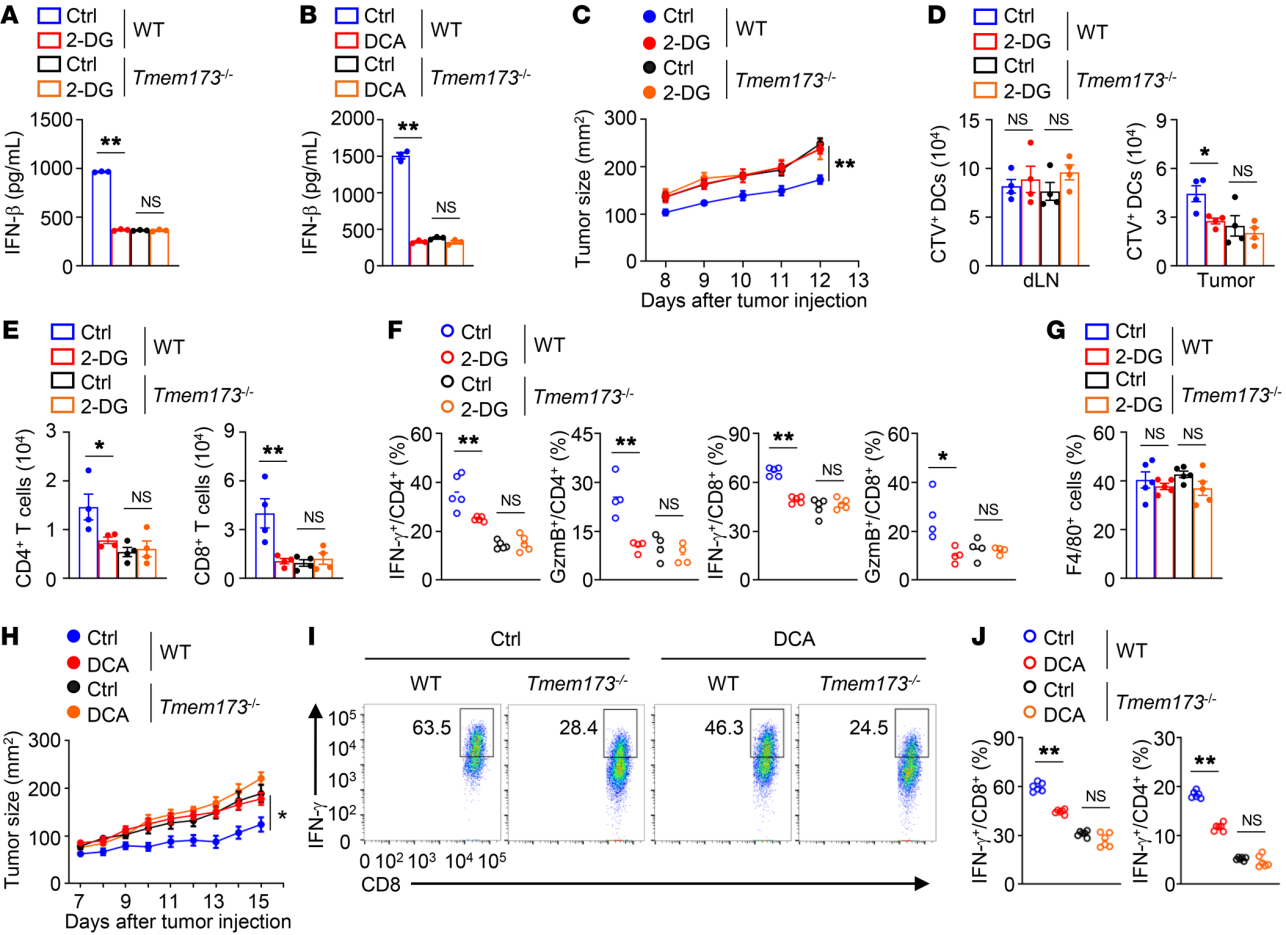
Glycolysis potentiates STING-dependent DC antitumor function. (**A** and **B**) ELISA analysis of BMDCs treated with 2-DG (1 mM; **A**) or DCA (10 mM; **B**) overnight and then stimulated with cGAMP for 8 hours. (**C**) MC38 tumor growth of WT mice transferred with cGAMP-stimulated BMDCs. BMDCs were labeled with CTV following 2-DG (1 mM) treatment for 8 hours. 2-DG–pretreated BMDCs were then stimulated with cGAMP for 4 hours. MC38 tumor-bearing WT mice were injected s.c. adjacent to the tumor with 2 × 10^6^ cGAMP-stimulated DCs on days 3 and 6 after tumor injection (*n =* 8). (**D**) The numbers of CTV^+^ DCs in the draining lymph nodes and tumors from mice from **C** on day 8 after tumor inoculation (*n =* 4). (**E**) The numbers of tumor-infiltrating CD4^+^ and CD8^+^ T cells from mice from **C** on day 14 after tumor inoculation (*n =* 4). (**F** and **G**) Flow cytometry analysis of tumor-infiltrating CD4^+^ and CD8^+^ T cells (**F**) or F4/80^+^ macrophages (**G**) from mice from **C** on day 14 after tumor inoculation. (**H**) Tumor growth of MC38 tumor-bearing WT mice transferred with cGAMP-stimulated DCs. BMDCs were pretreated with DCA (10 mM) for 8 hours and then stimulated with cGAMP for 4 hours. MC38 tumor-bearing WT mice were injected s.c. adjacent to the tumor with 2 × 10^6^ cGAMP-stimulated DCs on days 3 and 6 after tumor injection (*n =* 8). (**I** and **J**) Flow cytometry analysis of tumor-infiltrating CD8^+^ and CD4^+^ T cells of the mice from **H**. Representative data are shown from 2 (**C**–**J**) and 3 (**A** and **B**) independent experiments. Data are shown as the mean ± SEM. Statistical analysis was performed using 1-way ANOVA (**A**, **B**, **D**–**G**, and **J**) and 2-way ANOVA (**C** and **H**); *P < 0.05; **P < 0.01.

To validate the contribution of STING to glycolysis-mediated DC antitumor activity, we stimulated WT and STING-deficient BMDCs (STING is encoded by *Tmem173*) with cGAMP in the presence of 2-DG. The absence of STING abolished cGAMP-induced IFN-β production, and 2-DG treatment did not further suppress IFN-β production in cGAMP-stimulated STING-deficient DCs (Figure 4A). Similarly, treatment with DCA did not alter IFN-β production in cGAMP-stimulated STING-deficient DCs (Figure 4B). Using an animal model of BMDC-based therapy, we confirmed the importance of STING activity to the enhancing effect of glycolysis on DC antitumor functions. After inoculating WT mice with MC38 tumor cells, we injected WT BMDCs and STING-deficient BMDCs pretreated with cGAMP in the presence or absence of 2-DG into the tumor-bearing mice. Compared with the untreated WT DCs, the untreated STING-deficient DCs were less potent in suppressing tumor growth (Figure 4C). Pretreatment of WT DCs or STING-deficient DCs with 2-DG did not alter the numbers of cGAMP-stimulated DCs in the draining lymph nodes (Figure 4D). Notably, pretreatment of WT DCs with 2-DG reduced the numbers of tumor-infiltrating cGAMP-stimulated DCs, CD4^+^ T cells, and CD8^+^ T cells and decreased the frequencies of tumor-infiltrating IFN-γ–producing and granzyme B–producing CD4^+^ and CD8^+^ T cells (Figure 4, D–F). In contrast, mice injected with STING-deficient DCs pretreated with or without 2-DG displayed no apparent differences in the numbers of tumor-infiltrating cGAMP-stimulated DCs, CD4^+^ T cells, and CD8^+^ T cells or the frequencies of tumor-infiltrating IFN-γ–producing and granzyme B–producing CD4^+^ and CD8^+^ T cells (Figure 4, D–F). Pretreatment of WT DCs or STING-deficient DCs with 2-DG did not alter the frequencies of tumor-infiltrating macrophages (Figure 4G). Parallel studies revealed that DCA pretreatment also impaired WT DC-mediated antitumor immunity, and antitumor activities were comparable in mice injected with STING-deficient DCs with or without DCA pretreatment (Figure 4, H–J), suggesting that the defect in STING activity contributes to glycolysis inhibitor-induced impairment of DC antitumor functions. Therefore, glycolysis potentiates STING-dependent DC antitumor functions.

### Glycolysis promotes STING signaling via glycolytic ATP production.

**Figure 5:**
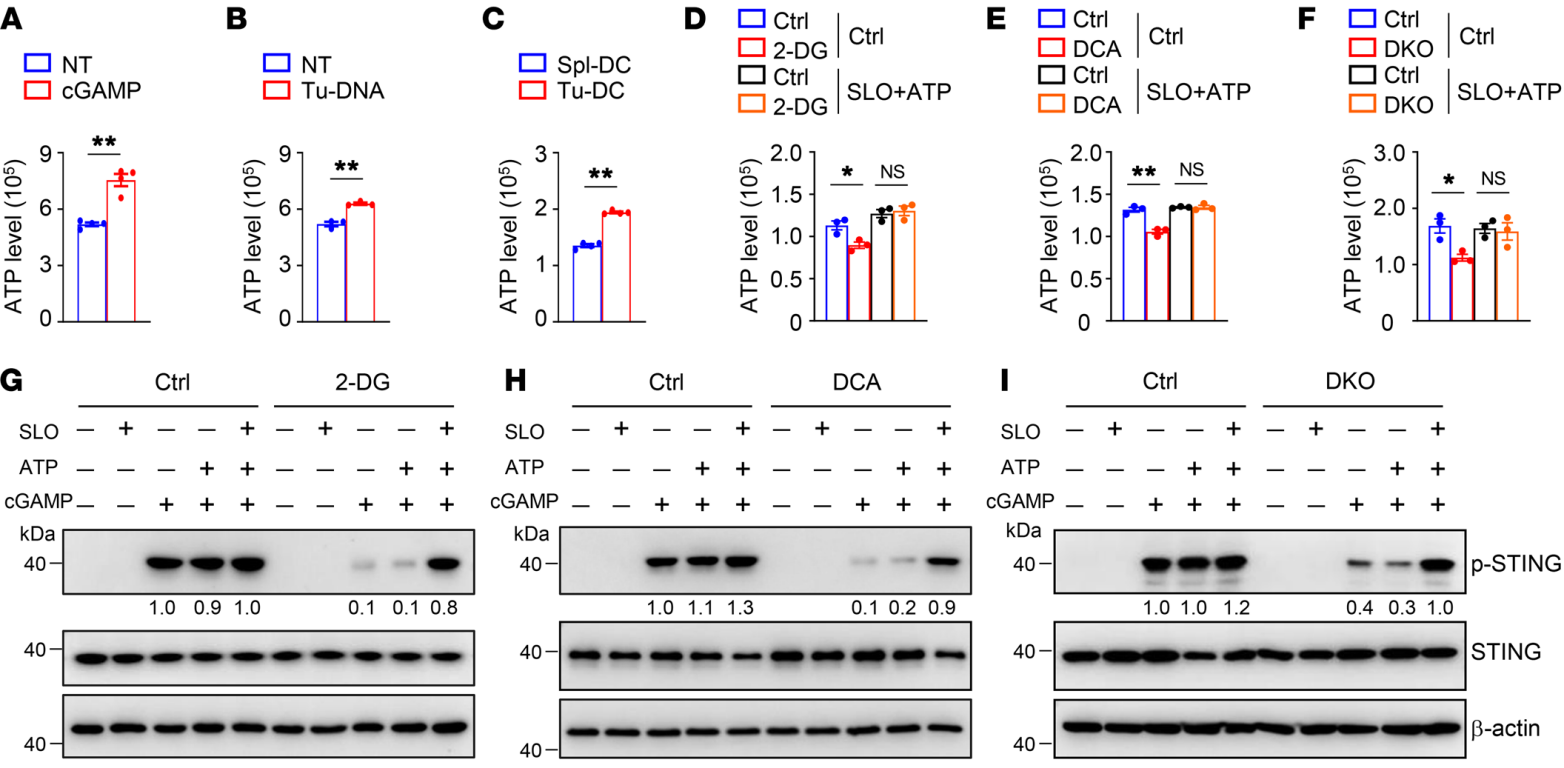
Glycolysis promotes STING signaling via glycolytic ATP production. (**A** and **B**) Intracellular ATP of BMDCs stimulated with 2 μg/mL cGAMP (**A**) or 40 μg/mL Tu-DNA (**B**) for 4 hours. (**C**) Intracellular ATP of splenic DCs (Spl-DC) and tumor-infiltrating DCs (Tu-DC) isolated from WT mice inoculated s.c. with MC38 colon cancer cells at 14 days. (**D** and **E**) Intracellular ATP of BMDCs stimulated with 2 μg/mL cGAMP in the presence of streptolysin-O (SLO) and ATP for 2 hours. BMDCs were pretreated with 2-DG (1 mM; **D**) or DCA (10 mM; **E**) overnight and subsequently stimulated as indicated. (**F**) Intracellular ATP of WT and *Ldha/b*-DKO (DKO) BMDCs stimulated with 2 μg/mL cGAMP in the presence of SLO and ATP for 2 hours. (**G** and **H**) Immunoblot analysis of indicated proteins in whole-cell lysates of BMDCs stimulated with 2 μg/mL cGAMP in the presence of SLO and ATP for 2 hours. BMDCs were pretreated with 2-DG (1 mM; **G**) or DCA (10 mM; **H**) overnight and subsequently stimulated as indicated. (**I**) Immunoblot analysis of indicated proteins in whole-cell lysates of WT and *Ldha/b*-DKO BMDCs stimulated with 2 μg/mL cGAMP in the presence of SLO and ATP for 2 hours. The numbers indicate the relative densities of indicated protein bands normalized to β-actin. Representative data are shown from 3 independent experiments. Data are shown as the mean ± SEM. Statistical analysis was performed using 2-tailed Student’s *t* test (**A**–**C**) and 1-way ANOVA (**D**–**F**); *P < 0.05; **P < 0.01.

Given that ATP production is tightly associated with glycolytic metabolism (21), we tested the cellular levels of ATP in activated DCs. Upon stimulation with cGAMP or tumor-derived DNA, the ATP production capacity in activated DCs was enhanced (Figure 5, A and B). In addition, tumor-infiltrating DCs were associated with elevated cellular ATP levels (Figure 5C). Treatment with 2-DG or DCA reduced ATP levels in activated DCs (Figure 5, D and E). Consistent with these observations, activated *Ldha*/*b*-DKO DCs exhibited a weaker ability to produce ATP (Figure 5F). Because treatment with ATP alone did not activate STING signaling or promote glycolysis (Supplemental Figure 5, A and B), we used streptolysin-O (SLO) to deliver exogenous ATP to DCs to investigate whether reduced ATP production accounts for the decrease in STING signaling. In the presence of ATP and SLO, ATP levels in activated DCs were considerably increased (Figure 5, D and E). Notably, glycolysis inhibitor treatment did not alter ATP levels in activated DCs under treatment with both ATP and SLO (Figure 5, D and E). Similarly, treatment with ATP and SLO increased ATP levels in activated *Ldha*/*b*-DKO DCs and abolished the difference in ATP levels between WT DCs and *Ldha*/*b*-DKO DCs (Figure 5F). Indeed, SLO-assisted intracellular delivery of ATP restored STING signaling activation in 2-DG– or DCA-treated activated DCs (Figure 5, G and H). Consistent with these results, the difference in cGAMP-induced STING phosphorylation between WT DCs and *Ldha*/*b*-DKO DCs was greatly reduced under treatment with both ATP and SLO treatment (Figure 5I). In contrast, treatment with SLO alone did not activate STING signaling or promote glycolysis (Figure 5, G–I, and Supplemental Figure 5, C and D). These data suggest that glycolysis promotes STING signaling via glycolytic ATP production.

### Glycolysis facilitates STING signaling in DCs from patients with NSCLC.

**Figure 6:**
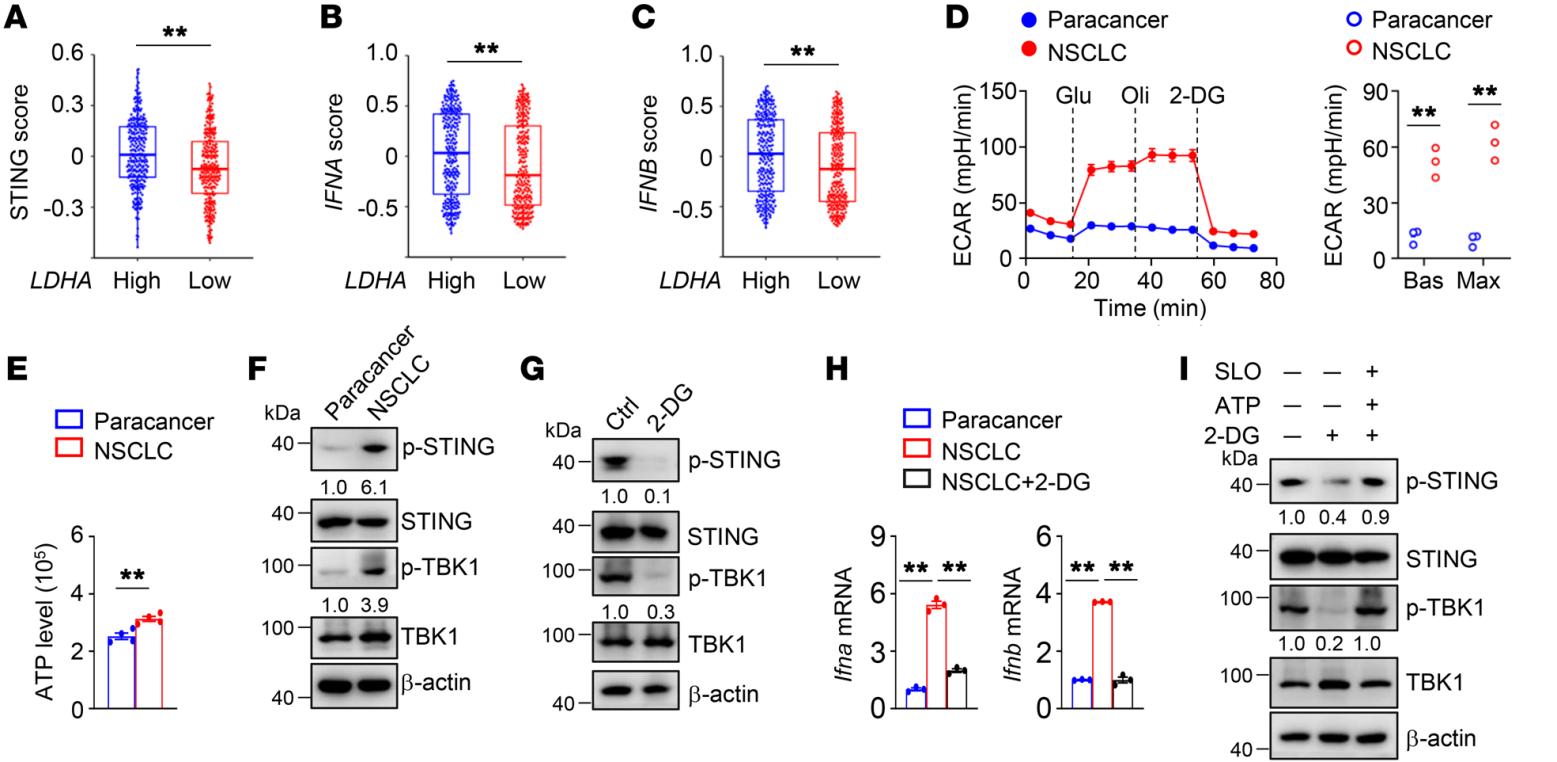
Glycolysis facilitates STING signaling in DCs from human NSCLC. (**A**–**C**) Correlation between LDHA transcripts and STING signature, including *STING* (**A**), *IFNA* (**B**), and *IFNB* (**C**), in TCGA data set of patients with lung cancer with low or high *LDHA* transcripts (*n =* 262). (**D**) ECAR of the freshly isolated DCs from the paracancerous tissue and NSCLC tissue under Bas or Max conditions. (**E**) Intracellular ATP of the freshly isolated DCs from the paracancerous tissue and NSCLC tissue. (**F**) Immunoblot analysis of indicated proteins in whole-cell lysates of the freshly isolated DCs from the paracancerous tissue and NSCLC tissue. (**G**) Immunoblot analysis of the freshly isolated NSCLC DCs treated with 2-DG (5 mM) for 8 hours. Ctrl, without 2-DG treatment. (**H**) qRT-PCR analysis of DCs isolated from the paracancerous tissue and NSCLC tissue and NSCLC DCs treated with 2-DG (5 mM) for 8 hours. (**I**) Immunoblot analysis of indicated proteins in whole-cell lysates of human NSCLC DCs that were pretreated with 2-DG (5 mM) for 6 hours and subsequently incubated in the presence or absence of ATP and SLO for 3 hours. The numbers indicate the relative densities of indicated protein bands normalized to β-actin. Data are representative of 3 independent experiments (**D**–**I**). Data are shown as the mean ± SEM. Statistical analysis was performed using a paired 2-sided Wilcoxon signed-rank test (**A**–**C**), 2-tailed Student’s *t* test (**D** and **E**), and 1-way ANOVA (**H**); **P < 0.01.

Given the potential importance of glycolysis in DC antitumor function, we examined the relationship between LDHA expression and the STING-dependent type I IFN signature. Interestingly, LDHA expression positively correlated with STING signaling, IFNA expression, and IFNB expression (Figure 6, A–C). Indeed, the levels of glycolytic rates in DCs from NSCLC tissue were higher than those in DCs from paracancerous tissue (Figure 6D). Consistent with these results, the levels of ATP and STING phosphorylation (at S366 in human STING) were increased in DCs from NSCLC tissue compared with DCs from paracancerous tissue (Figure 6, E and F). We next treated freshly isolated NSCLC DCs with 2-DG to confirm the involvement of glycolysis in STING-dependent type I IFN signaling in NSCLC tissue. As expected, STING phosphorylation and type I IFN induction were reduced in NSCLC DCs after 2-DG treatment (Figure 6, G and H). Importantly, SLO-assisted intracellular delivery of ATP restored STING phosphorylation in 2-DG–treated NSCLC DCs (Figure 6I). Collectively, these results indicate that the elevated glycolysis in tumor-infiltrating DCs facilitates STING signaling in human NSCLC.

### STING signaling promotes glycolysis and establishes a positive feedback loop.

**Figure 7:**
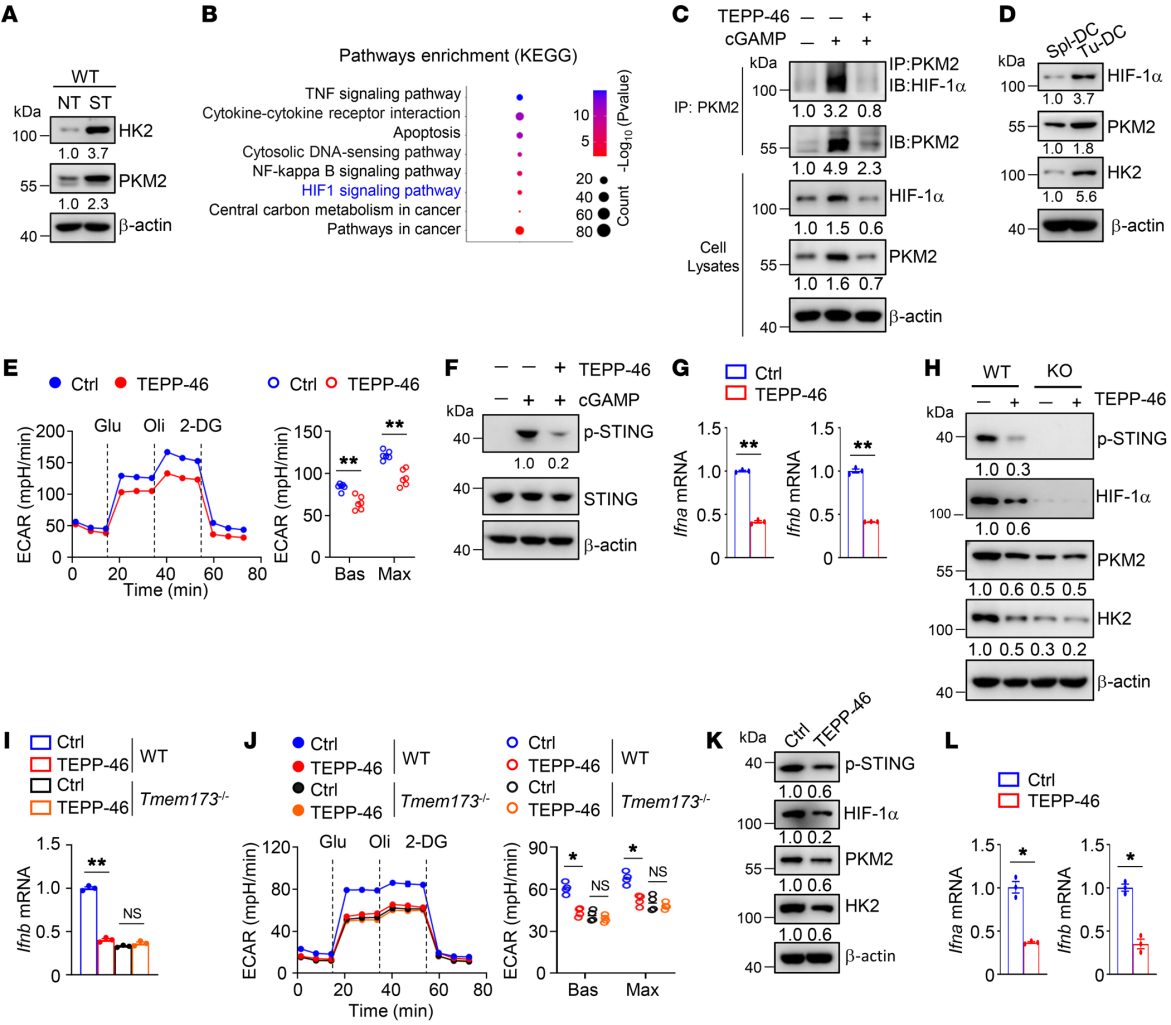
STING signaling promotes glycolysis and enables a positive feedback circuitry. (**A**) Immunoblot analysis of indicated proteins in whole-cell lysates of BMDCs stimulated with 2 μg/mL cGAMP for 4 hours. (**B**) Functional enrichment analysis of KEGG pathways in BMDCs stimulated with 2 μg/mL cGAMP for 4 hours. (**C**) Immunoprecipitation assays using indicated antibodies in BMDCs stimulated with 2 μg/mL cGAMP in the presence or absence of TEPP-46 (100 μM) for 4 hours. (**D**) Immunoblot analysis of indicated proteins in whole-cell lysates of splenic DCs (Spl-DC) and tumor-infiltrating DCs (Tu-DC) isolated from MC38 tumor-bearing WT mice on day 14 after tumor inoculation. (**E**–**G**) ECAR (**E**), immunoblot analysis (**F**), and qRT-PCR analysis (**G**) of BMDCs stimulated with 2 μg/mL cGAMP in the presence or absence of TEPP-46 (100 μM) for 4 hours. (**H**–**J**) Immunoblot analysis (**H**), qRT-PCR analysis (**I**), and ECAR (**J**) of tumor-infiltrating DCs from WT and *Tmem173^–/–^* (KO) mice i.p. injected with 50 mg/kg TEPP-46 on days 3, 5, and 7 after MC38 tumor cell inoculation. Ctrl, without TEPP-46 injection. (**K** and **L**) Immunoblot analysis (**K**) and qRT-PCR analysis (**L**) of human NSCLC DCs treated with TEPP-46 (100 μM) for 8 hours. The numbers indicate the relative densities of indicated protein bands normalized to β-actin. Data are representative of 3 independent experiments (**A** and **C**–**L**). Data are shown as the mean ± SEM. Statistical analysis was performed using 1-way ANOVA (**I** and **J**) and 2-tailed Student’s *t* test (**E**, **G**, and **L**); *P < 0.05; **P < 0.01.

We found that key glycolytic enzymes, such as hexokinase 2 (HK2) and pyruvate kinase M2 (PKM2), were greatly accumulated in cGAMP-stimulated WT DCs (Figure 7A), an effect that was responsible for the extensive enhancement of glycolysis in activated WT DCs. However, STING-deficient DCs stimulated with or without cGAMP exhibited no apparent difference in HK2 and PKM2 protein levels (Supplemental Figure 6A). Consistent with this finding, cGAMP stimulation did not increase the glycolytic rates or ATP levels in STING-deficient DCs (Supplemental Figure 6, B and C). Furthermore, stimulation with tumor-derived DNA did not change the glycolytic rates or ATP levels in STING-deficient DCs (Supplemental Figure 6, D and E). These results suggest that STING signaling controls glycolysis-related gene expression.

To determine the mechanistic link between glycolysis and STING signaling, we performed RNA-Seq using WT BMDCs stimulated with cGAMP. Functional pathway enrichment analysis revealed that cGAMP stimulation significantly affected the pathways involving cytosolic DNA sensing and central carbon metabolism (Figure 7B). Importantly, cGAMP stimulation upregulated the expression of HIF-1α–related genes and increased the protein level of HIF-1α (Figure 7, B and C, and Supplemental Figure 7A), which was responsible for the induction of glycolytic enzymes. In contrast, cGAMP stimulation did not alter the HIF-1α protein level in STING-deficient DCs (Supplemental Figure 7B), suggesting that HIF-1α–mediated induction of glycolytic enzymes relies on STING signaling. Notably, both PKM2 and HIF-1α accumulated in cGAMP-stimulated WT DCs (Figure 7, A and C). In addition, HIF-1α, HK2, and PKM2 were greatly accumulated in tumor-infiltrating DCs (Figure 7D). Because PKM2 is a critical modulator that regulates HIF-1α stability and activity (22), we examined the interplay between PKM2 and HIF-1α using the small-molecule drug TEPP-46, which can block the interaction between HIF-1α and PKM2 (22). As expected, TEPP-46 treatment inhibited the association of PKM2 with HIF-1α and attenuated the induction of HIF-1α and PKM2 (Figure 7C). Furthermore, treatment with TEPP-46 diminished glycolysis, STING phosphorylation, and type I IFN induction in cGAMP-stimulated WT DCs (Figure 7, E–G). Consistent with these results, the absence of HIF-1α abolished PKM2 accumulation, STING phosphorylation, type I IFN induction, and glycolysis in cGAMP-stimulated DCs (Supplemental Figure 7, C–F). HIF-1α–knockdown DCs were less potent in suppressing tumor growth and inducing tumor-infiltrating IFN-γ–producing T cells (Supplemental Figure 7, G and H). Importantly, TEPP-46 treatment attenuated the expression of HIF-1α, HK2, and PKM2, an diminished glycolysis, STING phosphorylation, and type I IFN induction in tumor-infiltrating WT DCs (Figure 7, H–J). However, TEPP-46 treatment did not alter the expression of HIF-1α and PKM2, the level of glycolytic activity, or the induction of type I IFN in tumor-infiltrating STING-deficient DCs (Figure 7, H–J). Notably, TEPP-46 treatment inhibited the induction of PKM2 and HIF-1α and resulted in defective activation of STING in NSCLC DCs (Figure 7, K and L). These findings suggest that DC-intrinsic STING activation accelerates HIF-1α–mediated glycolysis and establishes a positive feedback loop.

## Discussion

Our results demonstrate that glycolysis drives STING signaling to promote DC-mediated antitumor immune responses. The contribution of glycolysis to STING-dependent DC antitumor immunity was best discerned using *Ldha*/*Ldhb* DC-conditional knockout mice. LDHA/LDHB deficiency inhibited glycolytic metabolism and ATP production, thus diminishing the STING-dependent type I IFN response and limiting DC antitumor functions. Consequently, SLO-assisted intracellular delivery of ATP restored STING signaling activation in LDHA/LDHB-deficient DCs. Notably, STING signaling controlled HIF-1α–mediated expression of glycolysis-related genes and established a positive feedback loop. Importantly, glycolysis facilitated STING signaling in DCs from human NSCLC tissues. Our work showed that the glycolytic pathway is essential for the STING-dependent antitumor activity of DCs and thus defines a critical metabolic mechanism of DC-intrinsic STING signaling regulation.

Aerobic glycolysis is a metabolic hallmark of activated DCs, but the connection between glycolytic metabolism and STING signaling remains elusive. Using systematic approaches in conjunction with metabolomics and transcriptomic analysis, we demonstrated that elevated glycolysis-derived ATP production in activated DCs is required for STING phosphorylation and enhances its functions to orchestrate type I IFN production. Prior studies have suggested that glycolytic metabolism augments ATP production, triggering a PI3K-centered positive feedback regulatory circuit and driving effector T cell responses (20, 21). Based on these findings, we rationalized that ATP accumulation via DC- and T cell–intrinsic glycolysis may be a potential mechanism for host tumor immune surveillance. Therefore, pharmacologically enhancing glycolysis-dependent ATP production is a promising strategy to strengthen antitumor immune responses.

Recent studies have showed interplay between glucose metabolites and innate immune sensing during immune activation in response to viral infections or tumors (15, 23). Glycolysis-derived lactate directly binds to the mitochondrial antiviral-signaling (MAVS) protein and suppresses its functions to orchestrate type I IFN production (15), indicating that lactate acts as a natural barrier to impede cytosolic RNA sensing. Lactate also disrupts DC-mediated tumor rejection in the TME (24, 25), whereas its potential role in cytosolic DNA sensing remains to be determined. ROS, the important metabolites of glucose metabolism, are involved in STING-dependent immune sensing (4, 26). ROS-mediated DNA oxidation enhances immune recognition and potentiates STING signaling in autoimmunity (26). DC-derived ROS stabilize SENP3 to boost STING activation in the TME (4). On the other hand, ROS production blocks STING-dependent type I IFN responses in DNA virus-infected macrophages (27, 28), indicating that ROS production in different cell types may have different roles in STING activation. In our study, we demonstrated that the glucose metabolite ATP promoted STING phosphorylation to initiate DC-mediated antitumor immunity, suggesting that ATP is indispensable for cytosolic DNA sensing. However, whether and how other glucose metabolites participate in STING signaling is still unclear. Therefore, the functional importance of different glucose metabolites in STING-dependent immune sensing remains to be further studied.

STING agonists and tumor-derived DNA can activate the STING signaling pathway in DCs (4, 29). Our results revealed that glucose metabolism was primed in DCs upon STING activation. Elevated aerobic glycolysis contributed to STING signaling activation, although the potential role of other types of glucose metabolism still needs to be defined. DC-intrinsic STING activation accelerated HIF-1α–mediated glycolysis and establishes a positive feedback loop. STING deficiency abolished the increases in the glycolytic rates and ATP production in activated DCs, thereby inhibiting DC antitumor immune responses. Consistent with these findings, STING signaling drives HIF-1α stabilization to increase glycolysis in macrophages during *Brucella abortus* infection (30). Additionally, STING agonists activate STAT3 to govern glycolysis in intestinal epithelial cells (31). Future studies are needed to examine the potential interplay between STING signaling and HIF-1α or STAT3 in tumor-infiltrating DCs.

In summary, our findings elucidate an essential function for glycolysis in DC-mediated antitumor immunity. Our study provides crucial molecular insight into how the crosstalk of energy metabolism and STING signaling regulates DC antitumor activity. These results offer an important paradigm and strategy for the improvement of DC-centric immunotherapies.

## Methods

### Mice.

C57BL/6 background *Ldha*-floxed mice and *Ldhb*-floxed mice were generated at Shanghai Model Organisms. *Ldha/b*-DKO mice were obtained by crossing *Ldha*-floxed and *Ldhb*-floxed mice with *Cd11c*-Cre mice (The Jackson Laboratory). C57BL/6 background *Tmem173^–/–^* mice were generated at Shanghai Model Organisms. Age- and sex-matched mice were used in our experiments. All mice were housed in a specific pathogen–free facility.

### Cell culture.

Human embryonic kidney HEK293T cells (ATCC), MC38 murine colon cancer cells (Kerafast), B16-F10 murine melanoma cells (ATCC), and murine DC line DC2.4 cells (Sigma-Aldrich) were cultured in vitro. HEK293T and MC38 cells were cultured in DMEM medium supplemented with 10% FBS, 100 units/mL penicillin plus streptomycin. B16-F10 murine melanoma cells and DC2.4 cells were cultured in RPMI 1640 medium supplemented with 10% FBS, 100 units/mL penicillin plus streptomycin. Mouse bone marrow cells were harvested from tibias and femurs, and red blood cells were lysed with 150 mM NH_4_Cl/10 mM NaHCO_3_/1 mM EDTA. Remaining cells were cultured in RPMI 1640 medium supplemented with 20% FBS, 100 units/mL penicillin plus streptomycin, 20 ng/mL Granulocyte-Macrophage Colony Stimulating Factor (PeproTech) for 7–9 days to obtain BMDCs.

### Patients and specimens.

Human NSCLC tissues and paracancerous tissues were obtained from patients with NSCLC at Shanghai Chest Hospital, Shanghai Jiao Tong University School of Medicine. Fresh tumor tissues and paracancerous tissues were digested with collagenase VIII and DNase I. Immune cells were enriched by density-gradient centrifugation. DCs were isolated using a Pan-DC Enrichment Kit (130-100-777, Miltenyi) for further experiments.

### Antibodies and reagents.

The antibody against LDHB (PA5-27505; 1:1,000 for Western blotting [WB]) was purchased from Invitrogen (Thermo Fisher Scientific). The antibody against LDHA (NBP1-48336; 1:1,000 for WB) was purchased from Novus Biologicals. Antibodies against phosphorylated STING (p-STING) (human, 19781; 1:1,000 for WB), p-STING (mouse, 72971; 1:1,000 for WB), STING (50494; 1:1,000 for WB), p-TBK1 (5483; 1:1,000 for WB), TBK1 (3504; 1:1,000 for WB), HK2 (2867; 1:1,000 for WB), PKM2 (4053; 1:1,000 for WB), and HIF-1α (36169; 1:300 for WB) were purchased from Cell Signaling Technology. Anti–β-actin (A2228; 1:5,000 for WB) was purchased from Sigma-Aldrich. The fluorochrome-conjugated antibodies for CD8 (53-6.7), CD4 (RM4-5), CD62L (MEL-14), CD44 (IM7), IFN-γ (XMG1.2), granzyme B (GB11), F4/80 (BM8), CD11c (N418), MHC I (H-2Kb), CD80 (B7-1), and PDCA1 (eBio927) were purchased from Thermo Fisher Scientific. SLO was purchased from Sigma-Aldrich.

### DC stimulation and treatment.

BMDCs were harvested for experiments in vitro. For further analysis, BMDCs were counted and seeded in cell culture plates followed by cGAMP or purified Tu-DNA stimulation, respectively. cGAMP and Tu-DNA were transfected using Tenfect DNA transfection reagent (TEYE Corporation) according to the manufacturer’s instructions. Tu-DNA was obtained from MC38 genomic DNA using a Blood & Cell Culture DNA Mini Kit (13323, QIAGEN). cGAMP was purchased from InvivoGen Inc. TEPP-46 and DMXAA were purchased from Selleck. Sodium oxamic acid was purchased from Adamas-beta. DCA and cyclodextrin were purchased from Sigma-Aldrich. Human DCs were freshly isolated from NSCLC tissues and paracancerous tissues and purified with a Pan-DC Enrichment Kit (130-100-777, Miltenyi). The purified human DCs were untreated or treated with 2-DG or TEPP-46 for 8 hours and then used for further experiments.

### Flow cytometry.

Single-cell suspension of lymphocytes was generated for flow cytometry. For surface markers analysis, cells were stained with indicated fluorochrome-conjugated antibodies in MACS buffer (PBS+2%FBS) for 30 minutes. To detect intracellular cytokine production, cells were stimulated with phorbol 12-myristate 13-acetate and ionomycin in the presence of monensin for 4 hours and then stained with indicated fluorochrome-conjugated antibodies.

### WB and IP.

The cell pellets were collected and dissolved in lysis buffer (50 mM Tris-Hcl, 150 mM NaCl, 1 mM EDTA, 1% Triton X-100, 10% glycerol, phosphatase inhibitors and proteinase inhibitor cocktail) on ice for 20 minutes. The cell lysates were centrifuged at 13,800*g* for 20 minutes. Collected supernatants were used for WB and IP experiments. For IP assay, supernatants were immunoprecipitated with the appropriate antibodies using protein G–agarose beads overnight.

### RNA extraction and qRT-PCR.

Total RNA was extracted using TRIzol Reagent (Invitrogen). cDNA was synthesized using an All-in-One First Strand cDNA synthesis kit (Novoprotein). qRT-PCR was performed using qPCR SYBR Green Master Mix (YEASEN) and the primers as listed: mouse primers, IFNa forward, TGACCTCAAAGCCTGTGTGATG; IFNa reverse, AAGTATTTCCTCACAGCCAGCAG; IFNb forward, AGCTCCAAGAAAGGACGAACAT; IFNb reverse, GCCCTGTAGGTGAGGTTGATCT; actin forward, CGTGAAAAGATGACCCAGATCA; actin reverse, CACAGCCTGGATGGCTACGT; Pdha forward, GTGAGAACAACCGCTATGGCATG; Pdha reverse, CGCAAACTTTGTTGCCTCTCGG; Pkm2 forward, CAGAGAAGGTCTTCCTGGCTCA; Pkm2 reverse, GCCACATCACTGCCTTCAGCAC; Hk2 forward, TGATCGCCTGCTTATTCACGG; Hk2 reverse, AACCGCCTAGAAATCTCCAGA; Ldha forward, GGGCTACAAGCATCTTGAGAG; Ldha reverse, GACACGTTGCACCTGACTG; Eno1 forward, TGCGTCCACTGGCATCTAC; Eno1 reverse, CAGAGCAGGCGCAATAGTTTTA; Aldoa forward, TGGCGAGACTACTACCCAAGG; Aldoa reverse, GGGGCGAGGGAGTATGTTTC; Pgk1 forward, ATGTCGCTTTCCAACAAGCTG; and Pgk1 reverse, GCTCCATTGTCCAAGCAGAAT; human primers, IFNa forward, TCGCCCTTTGCTTTACTGAT; IFNa reverse, GGGTCTCAGGGAGATCACAG; IFNb forward, AAACTCATAGCAGTCTGCA; IFNb reverse, AGGAGATCTTCAGTTTCGGAGG; actin forward, GTCCTCTCCCAAGTCCACAC; and actin reverse, GGGAGACCAAAAGCCTTCAT.

### Bulk RNA-Seq.

BMDCs were untreated or stimulated with cGAMP for 4 hours in vitro. For tumor-infiltrating DC sequencing, the DCs were purified from spleens and tumor tissues of MC38 tumor-bearing WT mice using the CD11c^+^ Dendritic Cell Isolation Kit (130-108-338, Miltenyi). Cellular RNA was extracted using TRIzol Reagent (Invitrogen) and followed by Illumina Nextseq500 (150 bp paired end reads). Gene set enrichment analysis (GSEA) analysis was performed by the GSEA software. KEGG pathway enrichment analysis was performed by OmicsBean software. The RNA-Seq data reported here are deposited in the Genome Sequence Archive (Genomics, Proteomics & Bioinformatics 2021) in National Genomics Data Center (Nucleic Acids Res 2022), China National Center for Bioinformation/Beijing Institute of Genomics, Chinese Academy of Sciences that are publicly accessible at https://ngdc.cncb.ac.cn/gsa (CRA007915 and CRA007918).

### ATP measurement.

The intracellular ATP level was measured by a Cell Titer-Glo (CTG) Luminescent Cell Viability Assay kit (Promega). In brief, cells were seeded in a 12-well plate. CTG reagents were added into each well. Then, the cell plate was mildly mixed on the shaker for 10 minutes. The ATP luminescence signals were subsequently measured by using SpectraMax i3.

### ATP supplementation.

Cells were washed with cold PBS and then suspended with ATP-free HBSS buffer. Then, the cells were seeded in cell plates and treated with SLO (0.8 μg/mL). One hour later, the cells were supplied with 1.25 mM ATP for another 2 hours. After treatment, the cells were stimulated with cGAMP for 2 hours. Finally, the cells were immediately lysed for ATP measurement and WB analysis.

### CRISPR/Cas9-mediated gene editing.

gRNAs were annealed with respective complement-reverse sequences and were cloned into lentiCRISPRv2 vector. Lentiviruses were produced by cotransfection of lentiCRISPRv2 vector with 2 packing plasmids, pMD2.G and psPAX2, into HEK293T cells. The lentivirus-containing supernatants were collected at 48 hours and 72 hours after transfection and mixed together. For the infection assay, DC2.4 cells were infected with lentivirus-containing supernatants in the presence of polybrene (10 μg/mL) for 12 hours and then changed with fresh medium for another 12 hours. Then, the produce was repeated. Transduced cells were subsequently subjected to antibiotic selection with puromycin (5 μg/mL) to select resistant cells.

### Seahorse assays.

BMDCs and DC2.4 cells were stimulated with cGAMP for 4 hours. Tumor-infiltrating DCs of MC38 tumor-bearing mice and human NSCLC tissues were isolated and purified. All of the cells were attached to culture plates at a density of 1 × 10^5^ cells per well. Extracellular acidification rate and oxygen consumption rate were measured by XF96 Extracellular Flux Analyzer (Seahorse Biosciences) according to the manufacturer’s instructions, in the presence of the following compounds: 10 mM glucose, 2.5 μM oligomycin, 50 μM 2-DG, 1 mM FCCP, and 0.5 μM rotenone/antimycin A (Agilent Technologies).

### Tumor models.

MC38 murine colon cancer cells were cultured in DMEM medium supplemented with 10% FBS, 100 units/mL penicillin plus streptomycin. B16-F10 murine melanoma cells were cultured in RPMI 1640 medium supplemented with 10% FBS, 100 units/mL penicillin plus streptomycin. MC38 or B16-F10 tumor cells were s.c. injected into sex-matched 6- to 12-week-old WT and *Ldha/b*-DKO mice (5 × 10^5^ cells per mouse). Each experimental group included at least 7 mice to obtain a large enough data set. Tumor growth (tumor size represented tumor growth) and mouse survival (tumor size reached 225 mm^2^ indicating tumor-induced lethality) were monitored. For division of CTV-labeled OT-I T cells cocultured with tumor-infiltrating DCs, tumor-infiltrating DCs were treated with OVA (1.25 μg/mL) for 8 hours and then cocultured with CTV-labeled OT-I T cells for 72 hours. For the BMDC-based therapy tumor model, MC38 tumor-bearing mice were transferred s.c. adjacent to the tumor with WT and *Tmem173^–/–^* BMDCs that were untreated or treated with 2-DG or DCA overnight and subsequently stimulated with cGAMP for 4 hours. For DMXAA injection tumor model, WT and *Ldha/b-*DKO tumor-bearing mice were i.p. injected with 500 μg DMXAA on day 7 after MC38 tumor cell inoculation. DMXAA was dissolved in sterile PBS containing 0.75% NaHCO_3_. For TEPP-46 in vivo treatment, WT and *Tmem173^–/–^* tumor-bearing mice were i.p. injected with 50 mg/kg TEPP-46 on days 3, 5, and 7 after MC38 tumor cell inoculation. At day 14 after tumor inoculation, mice were sacrificed for further experiments. TEPP-46 was dissolved in PBS containing 40% cyclodextrin.

### Statistics.

All data are presented as the mean ± SEM. Statistical analysis were performed using GraphPad Prism 8.0 software. Significant differences were assessed by 2-tailed unpaired Student’s *t* test or 1-way or 2-way ANOVA statistical analyses. Survival curves were analyzed by log-rank (Mantel-Cox) test. The difference of STING signal score, IFNA score, and IFNB score between patients with high and low LDHA expression was assessed by Wilcoxon signed-rank test. GSEA analysis was performed using GSEA software (GSEA 4.1.10, https://www.gsea-msigdb.org/gsea/msigdb/). KEGG pathway enrichment analysis was performed using OmicsBean software. The statistical parameters are present in the figures and figure legends. Significance was set as *P <* 0.05.

### Study approval.

Human NSCLC tissues and paracancerous tissues were obtained from patients with NSCLC at Shanghai Chest Hospital, Shanghai Jiao Tong University School of Medicine. Informed consent was obtained from each patient. The study protocol was approved by the Clinical Research Ethics Committee of the Shanghai Chest Hospital and complied with all relevant ethical regulations. All animal experiments were in accordance with protocols approved by the Institutional Animal Care and Use Committee of Shanghai Jiao Tong University School of Medicine.

## Author contributions

ZH performed the experiments, analyzed the data, and wrote the paper. XY, RD, BL, CG, XWP, QH, YZ, and JW helped with mouse experiments and flow cytometry and analyzed the data. XGC, JS, and QZ designed the experiments, interpreted the results, wrote the paper, and oversaw the research project.

## Supplementary Material

Supplemental data

## Version 1 02/23/2023

In-Press Preview

## Version 2 04/03/2023

Electronic publication
