# Supplementary material for: Glycolysis drives STING signaling to facilitate dendritic cell antitumor function
Source: J Clin Invest. 2023 Apr 3;133(7):e166031. doi: 10.1172/JCI166031 (PMC10065062; doi:10.1172/JCI166031)
Supplement: Supplemental data [file jci-133-166031-s153.pdf]

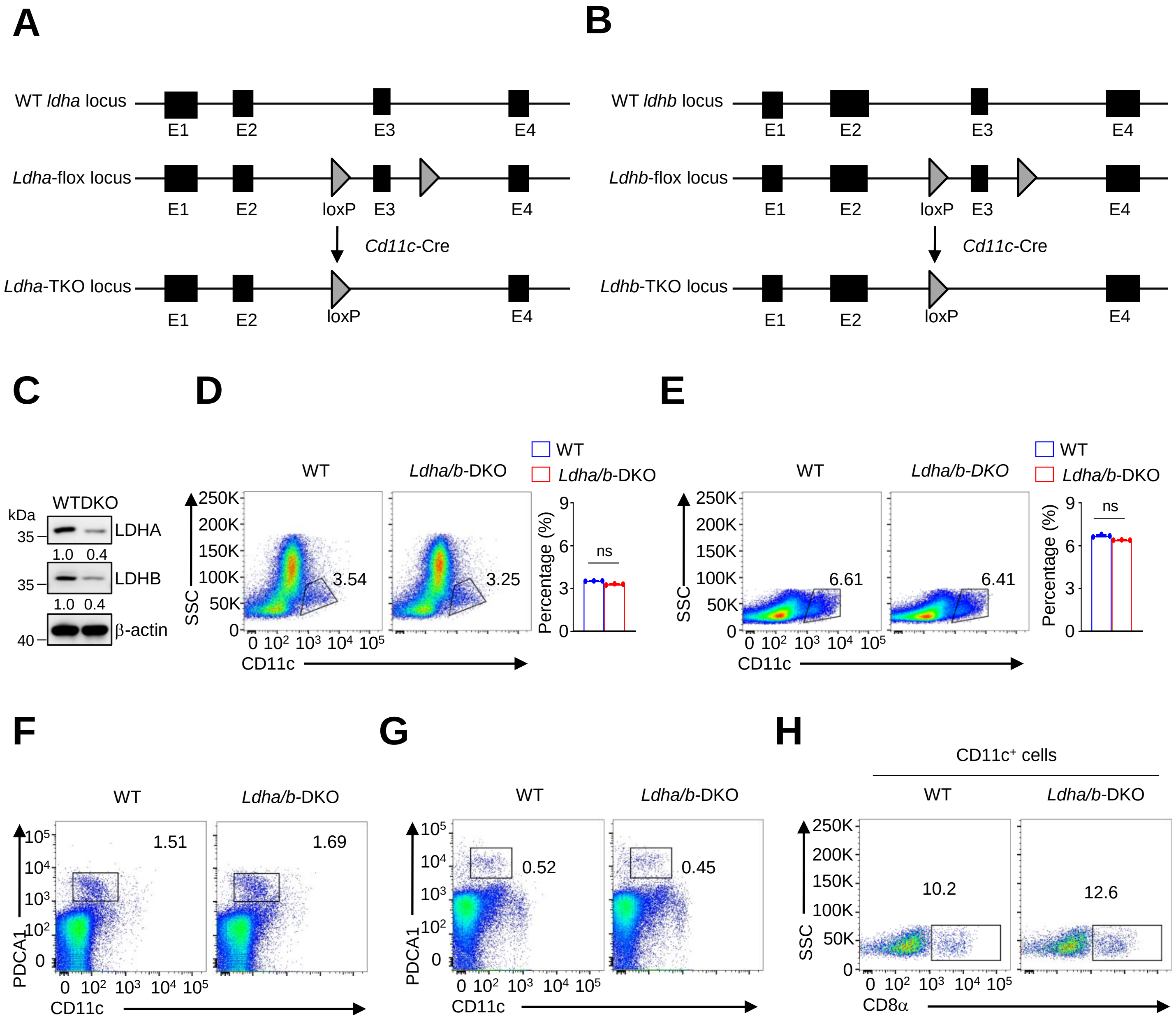

Supplementary Figure 1. LDHA and LDHB are not required for DC development. (**A** and **B**) Generation of *Ldha*-flox (**A**) and *Ldhb*-flox (**B**) mice. (**C**) Immunoblot analysis of indicated proteins in whole-cell lysates of WT and DKO BMDCs. *Ldha*$^{fl/fl}$*Ldhb*$^{fl/fl}$ mice were crossed with *Cd11c*-Cre mice to obtain *Ldha*$^{fl/fl}$*Ldhb*$^{fl/fl}$ (designated wild-type (WT)) and *Ldha*$^{fl/fl}$*Ldhb*$^{fl/fl}$ *Cd11c*-Cre (designated double knockout (DKO)) mice. (**D** and **E**) Flow cytometry analysis of the percentage of CD11c^{+} DCs in the bone marrow (**D**) or spleen (**E**). (**F** and **G**) Flow cytometry analysis of the percentage of plasmacytoid DC (CD11c^{+}PDCA1^{+}) in the bone marrow (**F**) or spleen (**G**). (**H**) Flow cytometry analysis of the percentage of CD8α^{+} DC (CD11c^{+} CD8α^{+}) in the spleen. Data are representative of three independent experiments. Normalized expression of indicated proteins to β-actin are displayed. Data are presented as mean ± SEM. Statistical analysis was performed using two-tailed Student's t test: ns, not statistically significant.

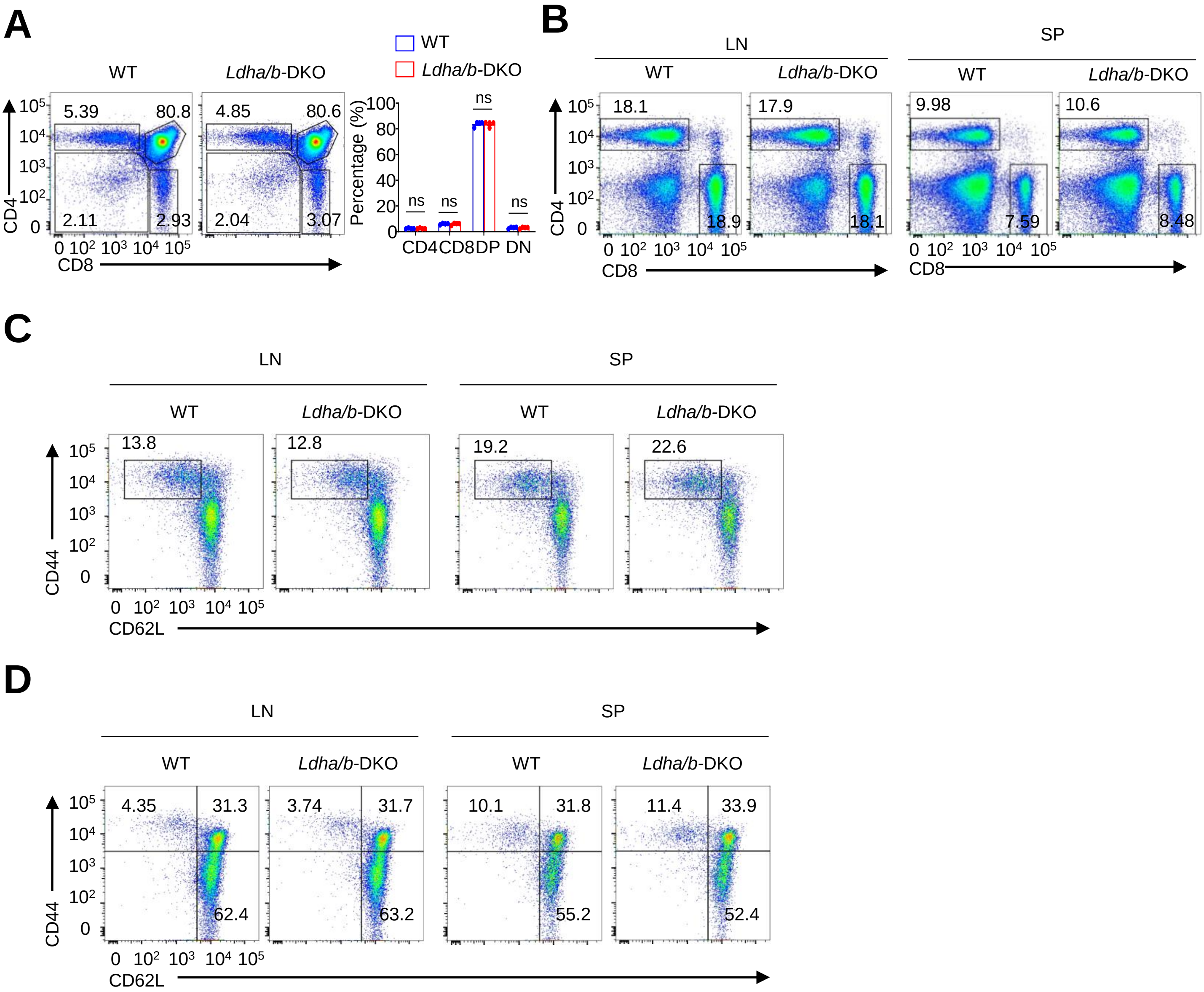

Supplementary Figure 2. LDHA and LDHB are not required for T cell development. Sex-matched 6-week-old WT and *Ldha/b*-DKO mice were sacrificed for flow cytometry analysis. (**A**) Flow cytometry analysis of the percentage of CD4 ($CD4^+$ $CD8^-$), CD8 ($CD4^-$ $CD8^+$), DP ($CD4^+$ $CD8^+$) and DN ($CD4^-$ $CD8^-$) T cells in the thymus. (**B**) Flow cytometry analysis of the percentage of $CD4^+$ and $CD8^+$ T cells in the lymph node (LN) and spleen (SP). (**C**) Flow cytometry analysis of CD44 and CD62L in $CD4^+$ T cells from lymph node (LN) and spleen (SP). (**D**) Flow cytometry analysis of CD44 and CD62L in $CD8^+$ T cells from lymph node (LN) and spleen (SP). Data are representative of three independent experiments. Data are presented as mean ± SEM. Statistical analysis was performed using two-tailed Student's t test: ns, not statistically significant.

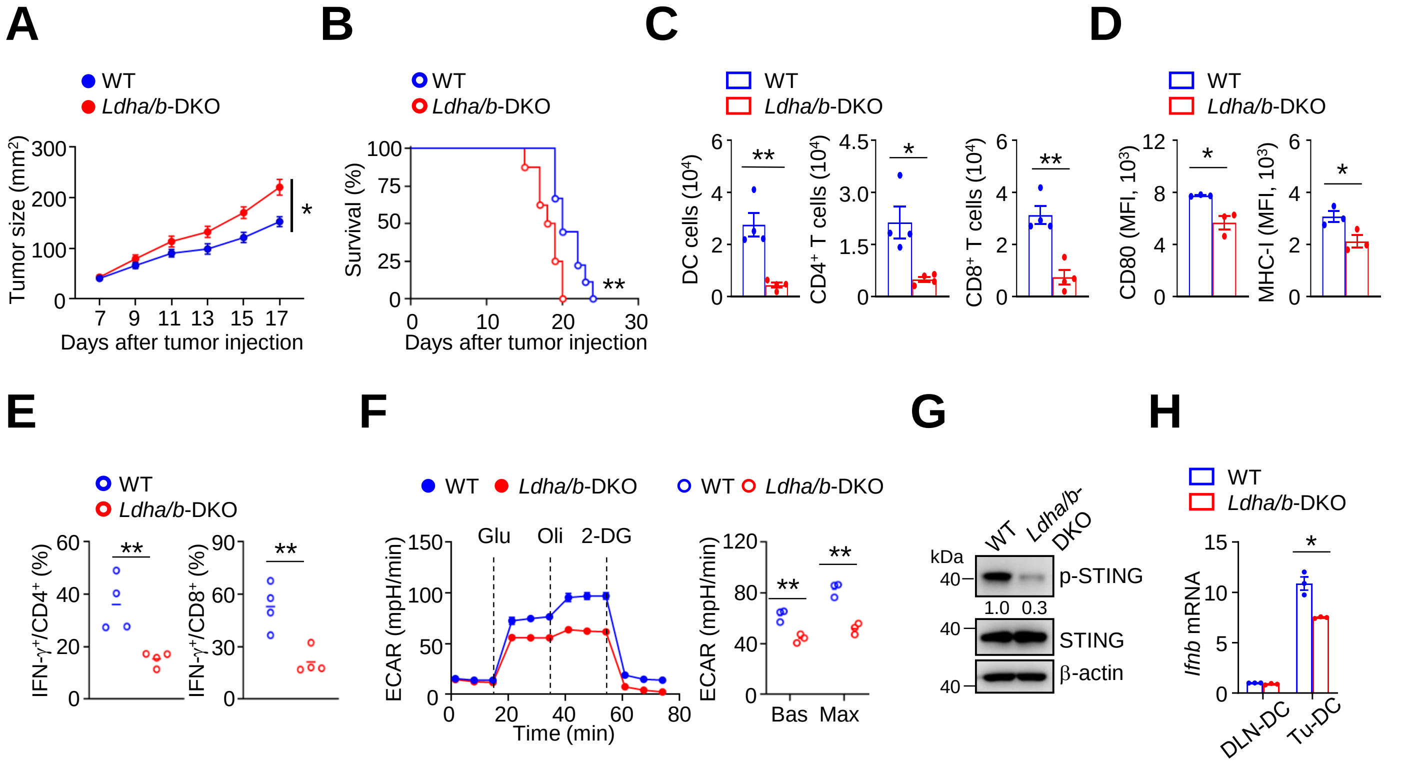

Supplementary Figure 3. Blockade of glycolysis inhibits DC antitumor function. (**A** and **B**) Tumor growth (**A**) and survival curves (**B**) of WT (n = 9) and *Ldha/b*-DKO mice (n = 8) inoculated subcutaneously (s.c.) with B16-F10 tumor cells. (**C**) The numbers of tumor-infiltrating DCs, $CD8^+$ and $CD4^+$ T cells of B16 tumor-bearing WT and *Ldha/b*-DKO mice at day 14 after tumor inoculation (n = 4). (**D**) Flow cytometry analysis of CD80 and MHC-I expression in the tumor-infiltrating DCs from B16 tumor-bearing WT and *Ldha/b*-DKO mice at day 14 after tumor inoculation (n = 3). **(E)** Flow cytometry analysis of IFN-γ-producing tumor-infiltrating $CD8^+$ and $CD4^+$ T cells from WT and *Ldha/b*-DKO mice inoculated s.c. with B16-F10 tumor cells at day 14 (n = 4). **(F)** ECAR of freshly isolated tumor-infiltrating DCs from WT and *Ldha/b*-DKO mice inoculated s.c. with B16-F10 cells at day 14 (n = 3) under Bas or Max conditions. **(G)** Immunoblot analysis of indicated proteins in whole-cell lysates of freshly isolated tumor-infiltrating DCs from WT and *Ldha/b*-DKO mice inoculated s.c. with B16-F10 cells at day 14. The numbers indicate the relative densities of indicated protein bands normalized to β-actin. (**H**) qRT-PCR analysis of freshly isolated tumor-infiltrating DCs from WT and *Ldha/b*-DKO mice inoculated s.c. with B16-F10 cells at day 14. Data are representative of three independent experiments. Data are presented as mean ± SEM. (**A**) was analyzed by using two-way ANOVA statistical analyses. (**B**) was analyzed by log-rank (Mantel-Cox) test. Statistical analysis (**C-F**, **H**) was performed using two-tailed Student's t test: *$p < 0.05$; **$p < 0.01$.

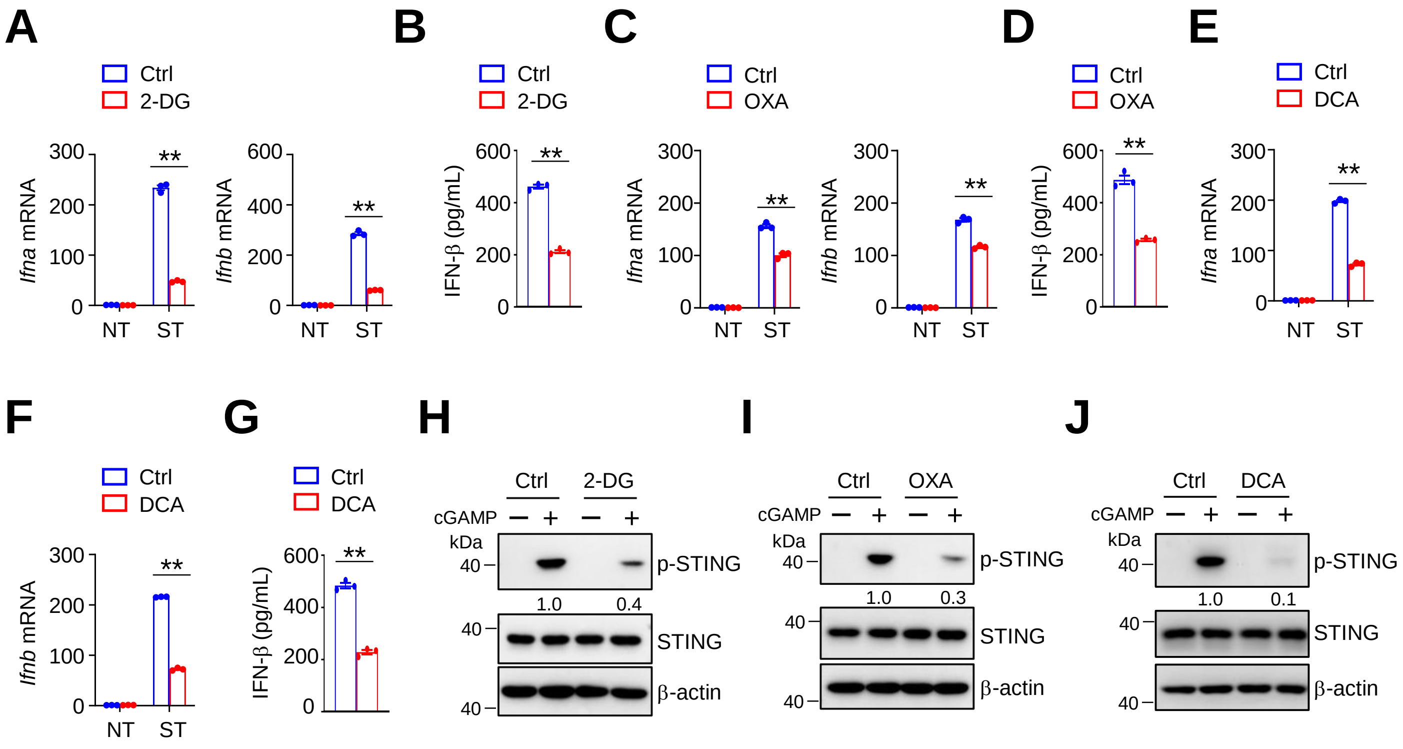

Supplementary Figure 4. Glycolysis inhibition impairs STING signaling pathway. BMDCs were treated with 2-DG (**A**, **B** and **H**; 1mM), Oxa (**C**, **D** and **I**; 20mM) and DCA (**E**, **F**, **G** and **J**; 10mM) overnight and subsequently stimulated with 2μg/mL cGAMP. (**A, C**, **E** and **F**) qRT-PCR analysis of indicated genes in BMDCs stimulated with cGAMP for 3 h. (**B**, **D** and **G**) ELISA analysis of IFN-β protein level in BMDCs stimulated with cGAMP for 8 h. (**H**, **I** and **J**) Immunoblot analysis of indicated proteins in whole-cell lysates of BMDCs stimulated with cGAMP for 4 h. NT, non-treated. ST, cGAMP treated. The numbers indicate the relative densities of indicated protein bands normalized to β-actin. Data are representative of three independent experiments. Data are presented as mean ± SEM. Statistical analysis was performed using two-tailed Student's t test: **$p < 0.01$.

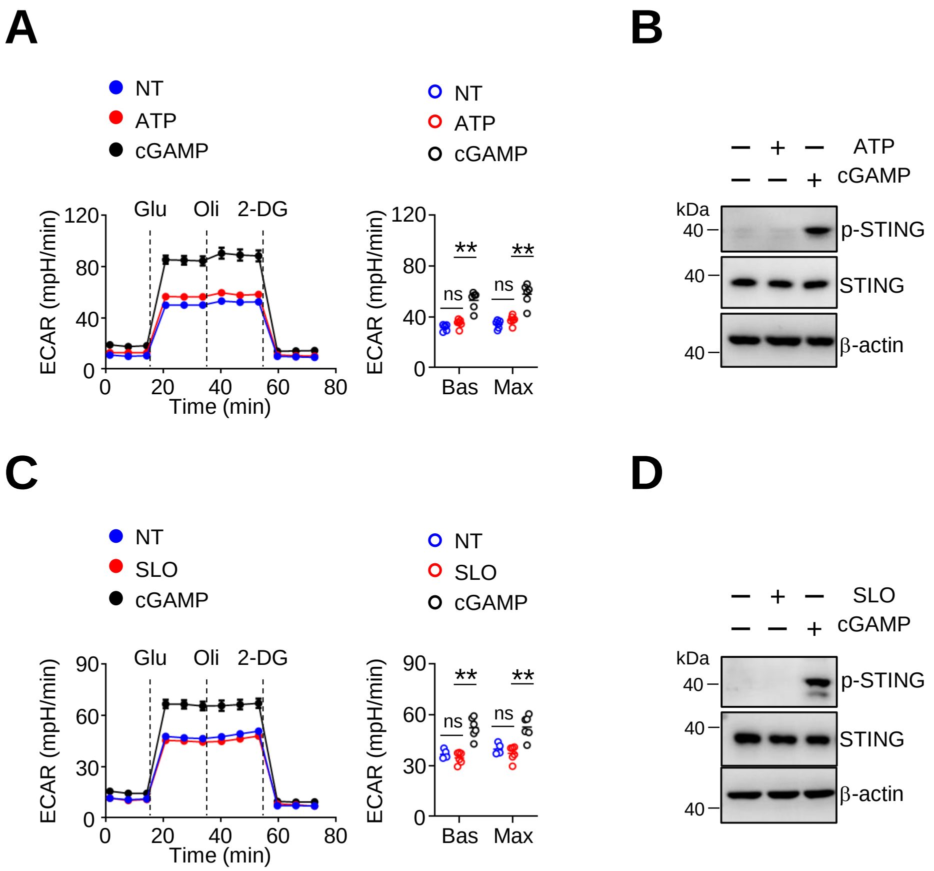

Supplementary Figure 5. Treatment with ATP or SLO alone does not promote glycolysis and STING activation in DCs. (**A**) ECAR under Bas or Max conditions of BMDCs treated with ATP or 2μg/mL cGAMP for 2 h. NT, non-treated. (**B**) Immunoblot analysis of indicated proteins in whole-cell lysates of BMDCs treated with ATP or 2μg/mL cGAMP for 4 h. (**C**) ECAR under Bas or Max conditions of BMDCs treated with SLO or 2μg/mL cGAMP for 2 h. NT, non-treated. (**D**) Immunoblot analysis of indicated proteins in whole-cell lysates of BMDCs treated with SLO or 2μg/mL cGAMP for 4 h. Data are representative of three independent experiments. Data are presented as mean ± SEM. Statistical analysis was performed using one-way ANOVA statistical analyses; ns, not statistically significant; $^{**}p < 0.01$.

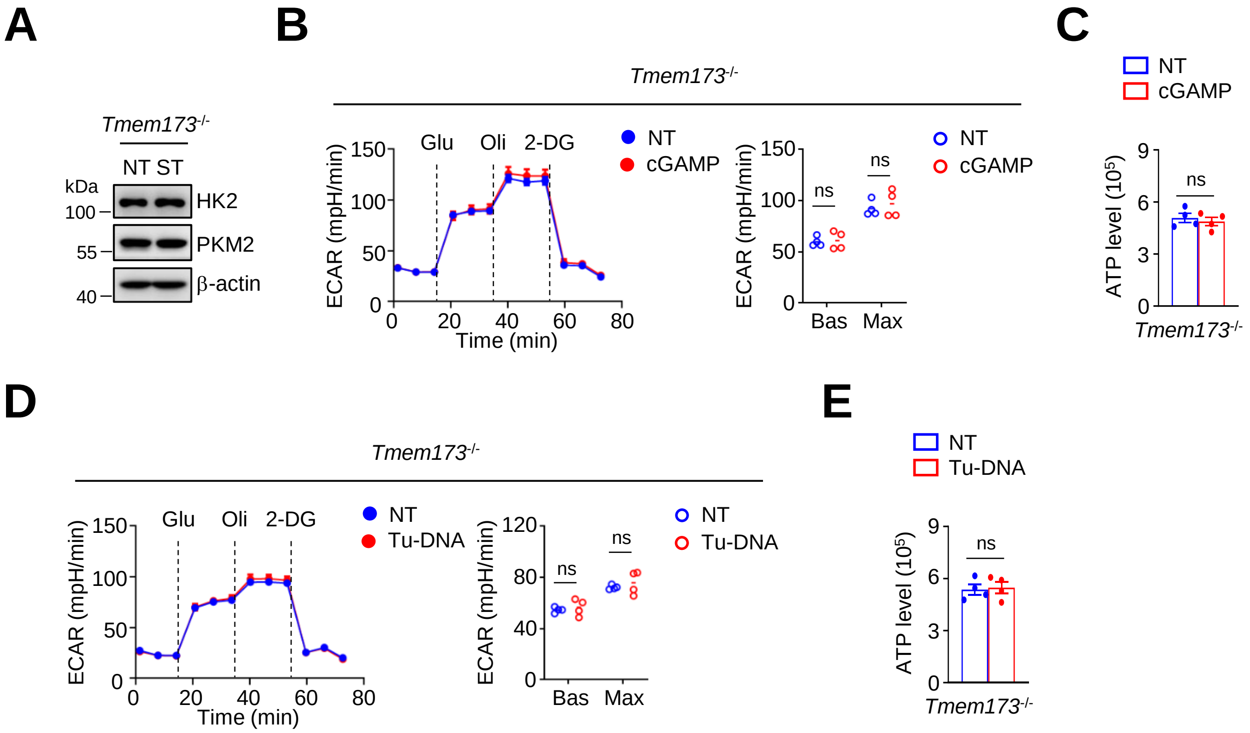

Supplementary Figure 6. STING signaling is required for cGAMP- and tumor DNA-induced glycolysis. (**A**) Immunoblot analysis of indicated proteins in whole-cell lysates of *Tmem173*$^{-/-}$ BMDCs stimulated with 2μg/mL cGAMP for 4 h. NT, non-treated. ST, cGAMP stimulated. (**B** and **D**) ECAR of *Tmem173*$^{-/-}$ BMDCs stimulated with 2μg/mL cGAMP (**B**) or 40μg/mL Tu-DNA (**D**) for 4 h. (**C** and **E**) Intracellular ATP of *Tmem173*$^{-/-}$ BMDCs stimulated with 2μg/mL cGAMP (**C**) or 40μg/mL Tu-DNA (**E**) for 4 h. Data are representative of three independent experiments. Data are presented as mean ± SEM. Statistical analysis was performed using two-tailed Student's t test: ns, not statistically significant.

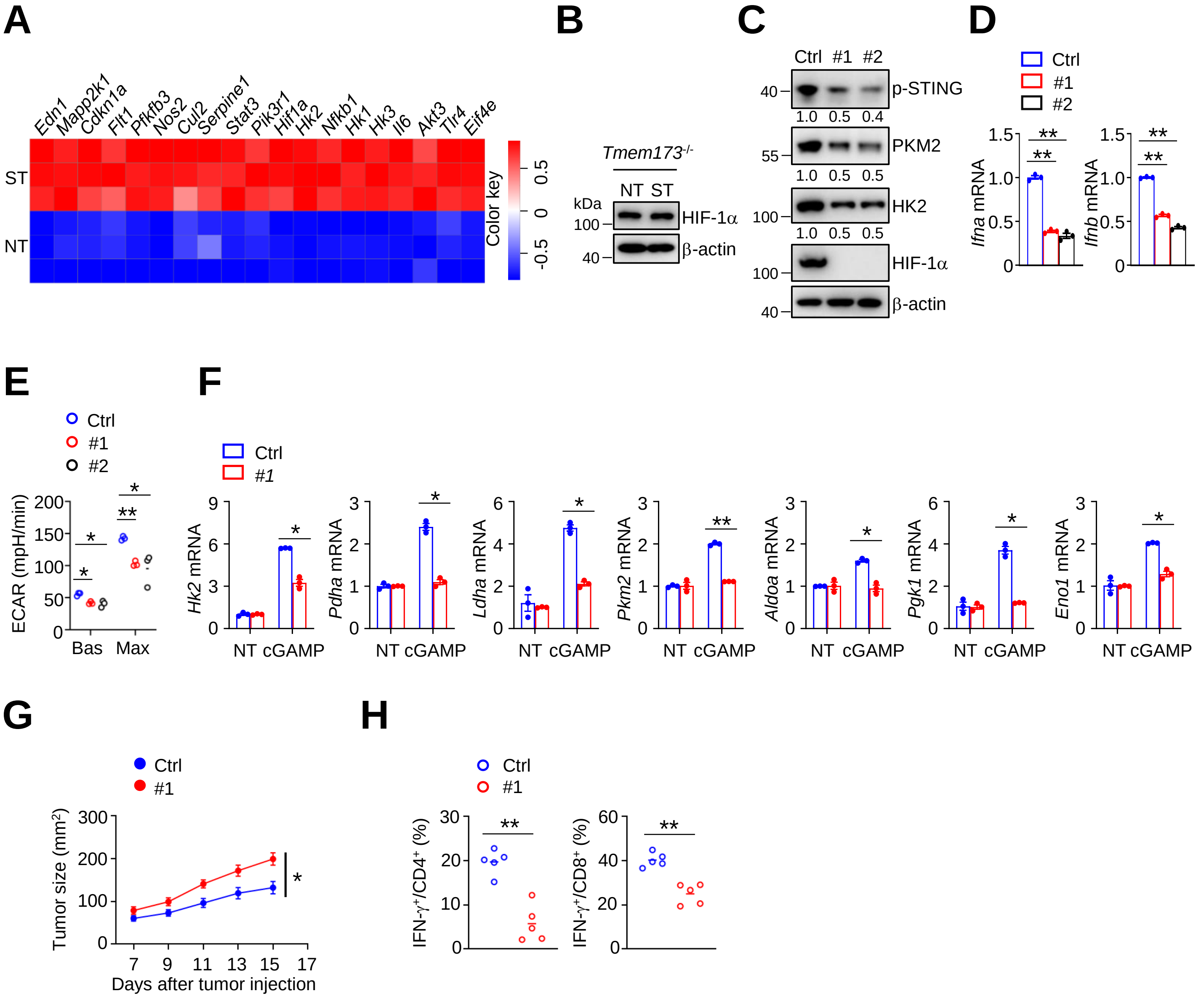

Supplementary Figure 7. DC-intrinsic STING activation accelerates HIF-1α signaling. (**A**) Heatmap analysis for HIF-1 signaling-related genes in WT BMDCs stimulated with 2μg/mL cGAMP for 4 h. (**B**) Immunoblot analysis of indicated proteins in whole-cell lysates of *Tmem173*$^{-/-}$ BMDCs stimulated with 2μg/mL cGAMP for 4 h. NT, non-treated. ST, cGAMP stimulated. (**C**-**E**) Immunoblot analysis (**C**), qRT-PCR analysis (**D**) and ECAR (**E**) of WT and CRISPR/Cas9-mediated *Hif1a*-knockout DC2.4 cells stimulated with 2μg/mL cGAMP for 4 h. Ctrl, WT DC2.4 cells; #1 and #2, *Hif1a*-knockout DC2.4 cells. (**F**) qRT-PCR analysis of glycolysis related genes in WT and CRISPR/Cas9-mediated *Hif1a*-knockout DC2.4 cells stimulated with 2μg/mL cGAMP for 4 h. Ctrl, WT DC2.4 cells; #1, *Hif1a*-knockout DC2.4 cells. (**G**) Tumor growth of MC38 tumor-bearing WT mice transferred with cGAMP-stimulated DCs. The WT (Ctrl) and *Hif1a*-knockdown BMDCs (#1) were stimulated with 2μg/mL cGAMP for 4 h. MC38 tumor-bearing WT mice were injected s.c. adjacent to the tumor with 2x10^6 cGAMP-stimulated DCs at day 3 after tumor cell inoculation (n = 10). **(H)** Flow cytometry analysis of IFN-γ-producing tumor-infiltrating CD8$^+$ and CD4$^+$ T cells from the tumor-bearing mice **(G)** (n = 5). The numbers indicate the relative densities of indicated protein bands normalized to β-actin. Data are representative of three independent experiments. Data are presented as mean ± SEM. (**F**, **H**) were performed using two-tailed Student's t test. (**D, E**) were analyzed by using one-way ANOVA statistical analyses. (**G**) was analyzed by using two-way ANOVA statistical analyses. *p < 0.05; **p < 0.01.
